# Construction of ceRNA Coexpression Network and Screening of Molecular Targets in Colorectal Cancer

**DOI:** 10.1155/2020/2860582

**Published:** 2020-04-21

**Authors:** Zhao Hui, Wang Zhanwei, Yang Xi, Liu Jin, Zhuang Jing, Han Shuwen

**Affiliations:** ^1^Department of Gastroenterology, Huzhou Central Hospital, Affiliated Central Hospital Huzhou University, 198 Hongqi Road, Huzhou, Zhejiang, China 313000; ^2^Department of Galactophore, Huzhou Central Hospital, Affiliated Central Hospital Huzhou University, 198 Hongqi Road, Huzhou, Zhejiang, China 313000; ^3^Department of Oncology, Huzhou Central Hospital, Affiliated Central Hospital Huzhou University, No. 198 Hongqi Road, Huzhou, Zhejiang Province, China 313000; ^4^Department of Pathology, Huzhou Central Hospital, Affiliated Central Hospital Huzhou University, No. 198 Hongqi Road, Huzhou, Zhejiang Province, China 313000; ^5^Graduate School of Nursing, Huzhou University, No. 1 Bachelor Road, Huzhou, Zhejiang Province 313000, China

## Abstract

**Objective:**

To screen some RNAs that correlated with colorectal cancer (CRC).

**Methods:**

Differentially expressed miRNAs, lncRNAs, and mRNAs between cancer tissues and normal tissues in CRC were identified using data from the Gene Expression Omnibus (GEO) database. The Kyoto Encyclopedia of Genes and Genomes (KEGG) pathway and protein-protein interactions (PPIs) were performed to do the functional enrichment analysis. And a lncRNA-miRNA-mRNA network was constructed which correlated with CRC. RNAs in this network were subjected to analyze the relationship with the patient prognosis.

**Results:**

A total of 688, 241, and 103 differentially expressed genes (diff-mRNA), diff-lncRNA, and diff-miRNA were obtained between cancer tissues and normal tissues. A total of 315 edges were obtained in the ceRNA network. lncRNA RP11-108K3.2 and mRNA ONECUT2 correlated with prognosis.

**Conclusion:**

The identified RNAs and constructed ceRNA network could provide great sources for the researches of therapy of the CRC. And the lncRNA RP11-108K3.2 and mRNA ONECUT2 may serve as a novel prognostic predictor of CRC.

## 1. Introduction

Colorectal cancer (CRC) is a common malignant tumor in the gastrointestinal tract [1]. The initial syndrome of CRC is not conspicuous; but with the development of the tumor, the patient will show changes in bowel habits, blood in the stool, diarrhea, alternating diarrhea and constipation, local abdominal pain, and other symptoms. Patients with advanced symptoms often show syndromes such as anemia and weakness [2]. CRC is the third most common malignant tumor around the world, and its mortality rate is extremely high [3]. Considering the great threat to human health of CRC, a series of new diagnostic and therapeutic methods have emerged. CRC is a complex disease involving the expression and structure of genes [4]. More and more studies have shown that miRNA can play a crucial aspect in cancer progression by regulating its related targets, including mRNA and lncRNA [5].

lncRNA is a noncoding RNA with a length of more than 200 nucleotides, which acts a pivotal part in abounding actions such as dose compensation effect, epigenetic regulation, cell differentiation regulation, and cell cycle regulation [6, 7]. It has been reported that upregulation of lncRNA FOXD3-AS1 suggests a lower survival rate in CRC patients. Experimental results showed that FOXD3-AS1 was overexpressed in CRC tissues and cells. Downregulation of FOXD3-AS1 expression in vitro can promote cell proliferation, invasion, and migration and promote apoptosis [8]. lncRNA-ATB and lncRNA-CCAT have strong accuracy in distinguishing CRC patients from healthy individuals [9]. miRNAs are endogenous noncoding RNAs with managerial actions, with a length of approximately 22 nucleotides that are involving posttranscriptional gene expression regulation in animals and plants [10]. miRNA-149 can be used as a target miRNA for identifying single bases in the serum of healthy and cancer patients. This method is direct and sensitive and can be used as an early diagnostic tool for colorectal cancer [11]. The expression of miR-139-5p was downregulated in the CRC cell line compared to the ordinary human colonic mucosal epithelial cell line NCM460. The study subsequently also demonstrated that overexpression of miR-139-5p in colon tumor cell lines significantly inhibited proliferation of cells *in vivo* and *in vitro*. The final study found that miR-139-5p regulates chronic inflammation by inhibiting NF-*κ*B activity, thereby inhibiting cell proliferation and invasion in CRC [12].

**Figure 1:**
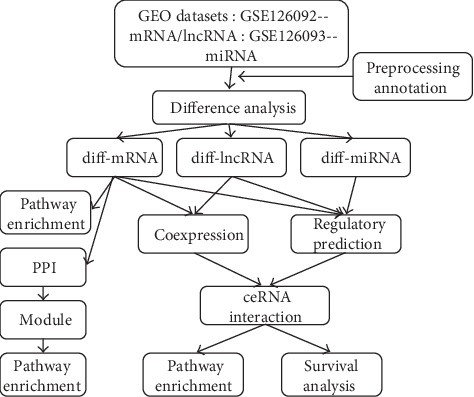
Diagram of bioinformatics analysis.

A series of studies have shown that miRNAs can silence gene by binding to mRNA, and lncRNA can regulate gene expression level relying on competitively binding miRNAs [13, 14]. At this study, a lncRNA-miRNA-mRNA coexpression network was constructed using two GEO datasets to screen RNAs that may be associated with CRC and supply a novel method for the diagnosis and therapy of CRC (Figure 1).

## 2. Materials and Methods

### 2.1. Data Collection

The lncRNA/mRNA profile data GSE126092 was downloaded from Gene Expression Omnibus (GEO) database (https://www.ncbi.nlm.nih.gov/gds/?term=). And the extracted data were produced by the platform of Agilent-074348 Human lncRNA v6 4 × 180 K (GPL21047, Probe Name Version). This dataset contained the data of lncRNA/mRNA expression profiles in 10 colorectal cancer (CRC) tissues and their corresponding normal-appearing tissues (NATs). The miRNA profile data GSE126093 was also extracted from GEO database. The data were produced by the platform of Exiqon miRCURY LNA microRNA array, 7th generation (GPL18058, miRBase v18, condensed Probe_ID version). This dataset contained ten cases of CRC tissues and their corresponding NATs.

### 2.2. Data Preprocessing and Screening of Differentially Expressed mRNA, lncRNA, and miRNA (diff-mRNA, diff-lncRNA, and diff-miRNA)

Limma package was used to preprocess the downloaded raw data and screen the differentially expressed mRNA, lncRNA, and miRNA. The preprocess process included background correction, normalization, and concentration prediction. The matrix data was combined with the chip platform annotation file to map the probe to the symbol. For multiple probes corresponding to an equal symbol, the final expression was decided by the mean of probes. Differential expression analysis of tumor vs. control was performed on the samples using the classical Bayesian test and corrected with Benjamini/Hochberg. *p* value < 0.05 and |log2 FC| > 2 were taken as the cut-off for the screening of diff-mRNA, diff-lncRNA, and diff-miRNA.

The heat maps of diff-mRNA, diff-lncRNA, and diff-miRNA were performed using heat map (version: 1.0.10, https://cran.r-project.org/web/packages/pheatmap/index.html).

### 2.3. Analysis of Protein-Protein Interactions (PPIs) in diff-mRNA

The PPIs of diff-mRNA analysis were performed using STRING (version: 10.0, https://www.string-db.org/) database with the score = 0.7, which was visualized by Cytoscape (version: 3.2.0, http://www.cytoscape.org/). Furthermore, based on MCODE (version 1.4.2, http://apps.cytoscape.org/apps/MCODE) with the score ≥ 10, the network modules were obtained and evaluated from the original PPI network.

### 2.4. Functional Enrichment Analysis

The Kyoto Encyclopedia of Genes and Genomes (KEGG) pathways for diff-mRNA, mRNAs in modules that were significantly clustered in PPIs, and mRNAs in ceRNA network were carried out, via the R package clusterProfiler (version 2.4.3, http://bioconductor.org/packages/3.2/bioc/html/clusterProfiler.html). In KEGG pathway enrichment analysis, *p* value < 0.05 and gene count > 2 were chosen as the cut-off criteria.

### 2.5. Prediction of diff-miRNA-diff-mRNA and diff-miRNA-diff-lncRNA

The miRWalk 2.0 (http://zmf.umm.uni-heidelberg.de/apps/zmf/mirwalk2/) was used to presume the target miRNAs of the diff-miRNA. The selected genes must have results in six of the eight databases (miRanda, miRWalk, miRDB, Pictar2, miRMap, RNA22, PITA, and TargetScan). miRanda (version: 3.3a, https://omictools.com/miranda-tool) was used to analyze potential diff-miRNA-diff-lncRNA binding sites with the score > 150 and energy<−20.

### 2.6. Coexpression Analysis of diff-lncRNA and diff-mRNA

The Pearson correlation coefficient (PCC) was calculated between lncRNA and mRNA. And the PPC > 0.95 and *p* value < 0.05 were considered to be meaningful correlation. Cytoscape was used to illustrate the coexpression network.

### 2.7. Survival Analysis

The colon cancer tumor samples from which mRNA, lncRNA, miRNA, and survival prognosis messages were extracted from the TCGA database, and the expression values and survival prognosis information of all node elements in the lncRNA-miRNA-mRNA network were extracted from the TCGA colon cancer samples. K-M survival curve was performed using Survival (version: 2.42-6, https://cran.r-project.org/web/packages/survival/index.html). *p* value < 0.05 was considered as the significant threshold.

## 3. Results

### 3.1. Clustering of Differentially Expressed RNAs in CRC Tissues

**Figure 2:**
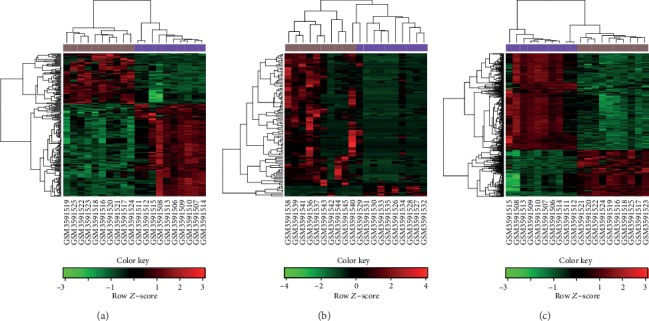
The heat maps of the differentially expressed lncRNAs, miRNAs, and mRNAs in CRC. The relative expression of the differentially expressed lncRNAs (a), miRNAs (b), and mRNAs (c), respectively, between the cancer tissues and normal tissues. Green indicates downregulated RNAs, and red indicates upregulated RNAs.

A total of 688, 241, and 103 differentially expressed mRNAs (diff-mRNAs), diff-lncRNAs, and diff-miRNAs were obtained, respectively, including 266 upregulated diff-mRNA, 462 downregulated diff-mRNA, 85 upregulated diff-lncRNA, 156 downregulated diff-lncRNA, 103 upregulated diff-miRNA, and 95 downregulated diff-miRNA. As shown in Figure 2, the two groups of samples could be separated significantly.

The top 10 expression of diff-mRNA was MMP7, LEMD1, CLDN1, SLCO1B3, REG1A, COL10A1, ETV4, COL11A1, APOBEC1, and LGR5. The top 10 expression of the following diff-lncRNA was CCAT1, LOC401585, AC123023.1, UCA1, EVADR, RP11-132A1.4, CTD-2008A1.3, SNAR-H, SNAR-C1, and SNAR-E. The top 10 expression of diff-miRNA was hsa-miR-31-5p, hsa-miR-224-5p, hsa-miR-1244, hsa-miR-188-5p, hsa-miR-764, hsa-miR-301b, hsa-miR-19b-1-5p, hsa-miR-3648, hsa-miR-452-3p, and hsa-miR-3157-3p. The details were shown in Supplementary tables 1, 2, and 3.

### 3.2. Screening the Cell Pathways with regard to Differentially Expressed mRNA in CRC

KEGG pathway analysis was performed to make a deep understanding between the diff-mRNA and cell pathways. The result indicated that all 266 upregulated diff-mRNAs were enriched in 10 pathways, including cell cycle, oocyte meiosis, progesterone-mediated oocyte, and cytokine-cytokine receptor interaction. And the 462 downregulated diff-mRNAs were enriched in 21 pathways, including hypertrophic cardiomyopathy, dilated cardiomyopathy, cGMP-PKG signaling pathway, and cAMP signaling pathway (Table 1).

### 3.3. PPI Network with regard to Differentially Expressed mRNAs in CRC

**Figure 3:**
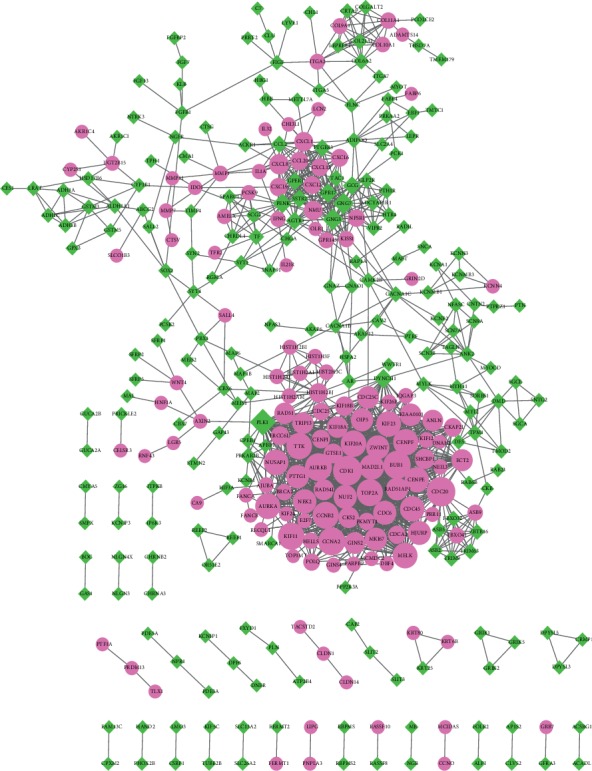
The protein-protein interaction network of differentially expressed mRNAs. The purple nodes represented the upregulated mRNAs, and the green nodes represented the downregulated mRNAs. The higher the degree value is, the larger the nodes.

**Figure 4:**
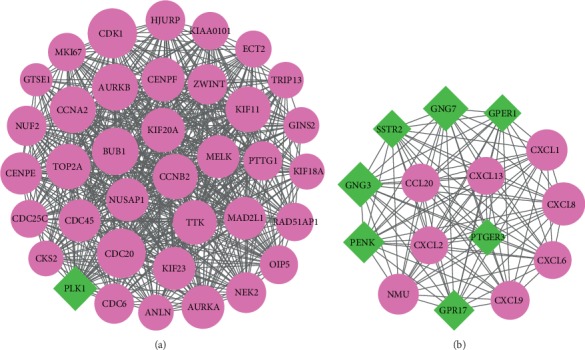
The protein-protein interaction network of differentially expressed mRNAs in module A (a) and module B (b). The purple nodes represented the upregulated mRNAs, and the green nodes represented the downregulated mRNAs. The higher the degree value is, the larger the nodes.

PPI network was constructed, which integrates large amount of known and predicted interactions of proteins. As shown in Figure 3, 354 nodes and 1,500 edges were included in the network. Module analysis was performed using MCODE, and two modules were obtained (module A and module B). Module A (score = 32.778) was constructed with 37 nodes and 590 edges, and module B (score = 15) was constructed with 15 nodes and 105 edges (Figures 4(a) and 4(b)). KEGG analysis was also performed. The result indicated that 37 diff-mRNAs in module A were enriched in 4 pathways, and 15 diff-mRNAs in module B were enriched in 8 pathways (Table 2).

### 3.4. miRNA-lncRNA/mRNA Prediction and lncRNA-mRNA Coexpression

The top 10 upregulated and all 8 downregulated diff-miRNAs were predicted using miRWalk 2.0 database. The edges were screened which the target genes were diff-mRNA. And a total of 308 edges of diff-miRNA-diff-mRNAs were obtained finally, including 15 diff-miRNAs and 177 diff-mRNAs. Thirty-nine edges of lncRNA-miRNA were obtained including 10 diff-miRNAs and 33 diff-lncRNAs. Furthermore, 143 target genes regulated by 10 miRNAs in the lncRNA-miRNA regulatory network were screened and coexpressed with 33 lncRNAs in the lncRNA-miRNA regulatory network. Based on the screening threshold, 77 edges of positive correlation lncRNA-mRNA were obtained.

### 3.5. lncRNA-miRNA-mRNA Network

According to the whole relationship of edges above, the lncRNA-miRNA-mRNA edges were further collated. A total of 315 edges were obtained, of which 77 lncRNA-mRNA positive correlation coexpression edges, 199 miRNA-mRNA regulatory edges, and 39 lncRNA-miRNA regulatory edges. And all the edges contained 186 nodes, 7 up-miRNAs, 3 down-miRNAs, 34 up-mRNAs, 109 down-mRNAs, 16 up-lncRNAs, and 17 down-lncRNAs.

**Figure 5:**
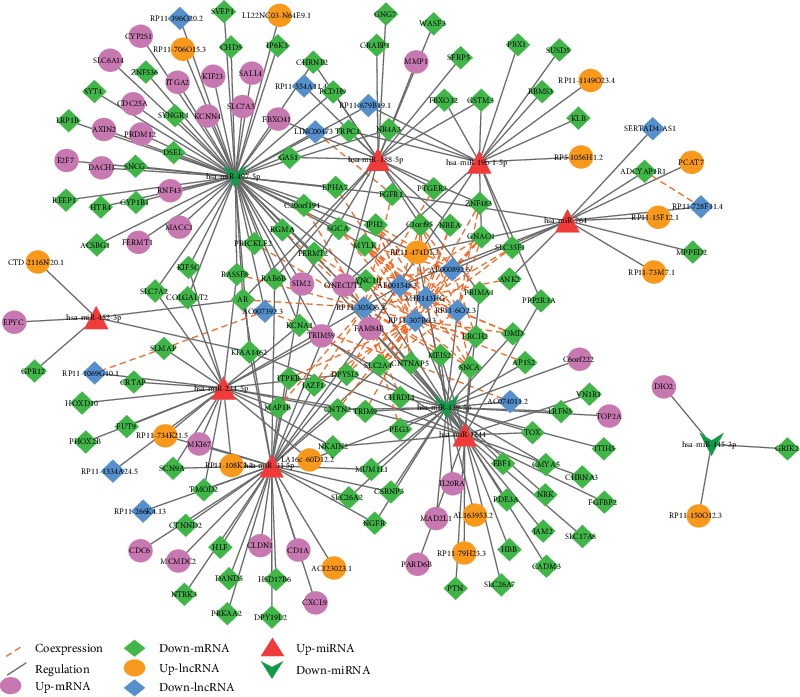
ceRNA network in CRC. The purple ellipse and the green diamond represented the up- and downregulated mRNAs, respectively. The orange ellipse and the blue diamond represented the up- and downregulated lncRNAs, respectively. The red triangle and the dark green arrow represented the up- and downregulated miRNAs, respectively. The red dotted line represented the coexpression relationships, and the black line represented the regulation relationships.

lncRNA-miRNA-mRNA network is shown in Figure 5, and the nodes in the network are shown in Supplementary Table 4. The diff-mRNAs in lncRNA-miRNA-mRNA network were analyzed using KEGG pathway enrichment analysis, of which 15 pathways (Table 3).

### 3.6. Differentially Expressed RNAs with regard to the Prognosis of CRC

**Figure 6:**
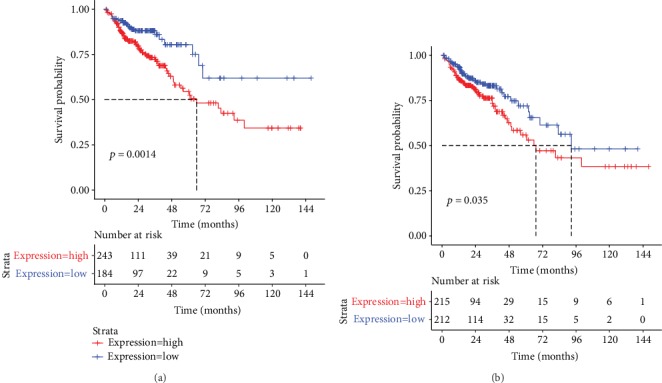
Survival curve with CRC containing differentially expressed RNAs. The mRNA, lncRNA, and miRNA expression levels and patient survival information from TCGA CRC data were used to plot the KM survival curve. The figure showed that the lncRNA RP11-108 K3.2 (a) and mRNA ONECUT2 (b) significantly correlated with prognosis of CRC.

All the nodes in Figure 6 were used to do the survival analysis, of which a lncRNA RP11-108K3.2 and one mRNA ONECUT2 correlated with prognosis.

## 4. Discussion

In this study, to screen for RNAs and pathways associated with CRC, a lncRNA-miRNA-mRNA expression network was constructed using two datasets downloaded from the GEO database. A total of 688 diff-mRNAs, 241 diff-lncRNAs, and 103 diff-miRNAs were identified at the present study. A functional enrichment analysis of these 688 diff-mRNAs revealed that they were mainly enriched in 21 pathways. A PPI network analysis was also performed on these diff-mRNAs. Finally, a ceRNA network was constructed and a survival analysis was performed on the nodes to obtain two prognostic-related RNAs. The expression levels of RP11-108K3.2 and ONECUT2 were both upregulated in CRC, and low levels were associated with better prognosis, suggesting that they may play a positive role in CRC.

lncRNA can participate in the initiation and development of many diseases by directly regulating proteins or indirectly regulating the target genes of related miRNA. The result of ceRNA showed that RP11-108K3.2 was regulated by hsa-miR-224-5p. Study has shown that the great expression level of RP11-108K3.2 correlated with low overall survival of CRC, and this result was consistent with our study [15]. In a study on lung adenocarcinoma, the researchers study 346 differentially expressed lncRNAs, of which RP11-108K3.2 is highly expressed in lung cancer tissues [16]. Furthermore, hsa-miR-224-5p is upregulated both in adenoma and in cancer tissues [17]. A study has been revealed that hsa-miR-224-5p could suppress the cell growth of two oral squamous cell carcinoma cell lines (SCC4, SCC15). However, when hsa-miR-224-5p and pcDNA3.1-CT-GFP-FTH1P3 are cotransfected, the growth inhibition of hsa-miR-224-5p on oral squamous cell carcinoma will be reversed [18]. In this study, the expressions of RP11-108K3.2 and hsa-miR-224-5p were both upregulated in CRC samples. This result indicates that lncRNA RP11-108K3.2 and hsa-miR-224-5p may play a same role as hsa-miR-224-5p and FTH1P3.

ONECUT2 is a member of the ONECUT2 transcription factor family that interacted with hsa-miR-139-5p, hsa-miR-188-5p, hsa-miR-19b-1-5p, hsa-miR-31-5p, and hsa-miR-497-5p in a ceRNA network in this study [19]. It has been reported that ONECUT2 acts a critical position on CRC gene network and is significantly associated with the development of cancer. Interference with endogenous ONECUT2 expression inhibited the CRC cell line SW620 cell migration [20]. A series of experiments have shown that hsa-miR-139-5p acts as a crucial part in colon tumor. Compared with normal tissues, the hsa-miR-139-5p expression level is significantly declined in colon tumor, and its expression level is significantly correlated with tumor stage. Subsequently, experiments have shown that the direct object of hsa-miR-139-5p is BCL2, and the expression of BCL2 is negatively correlated with the expression of hsa-miR-139-5p. The tumor metastasis and drug sensitivity of CRC could be diminished by hsa-miR-139-5p targeting the BCL2 pathway [21]. In the present study, hsa-miR-139-5p was interacted with ONECUT2, and its expression level was negatively connected with the expression level of the later one. These two RNAs may have a similar mechanism with hsa-miR-139-5p and BCL2. hsa-miR-31-5p as an infrequently expressed miRNA was associated with a stage when assessing CRC cases. The upregulated hsa-miR-31-5p makes a more advanced disease stage more likely than a lower disease stage [22]. At this research, the expression level of hsa-miR-31-5p was higher in CRC tissues than in control (logFC = 9.153176). In addition to being significantly upregulated in CRC tissues, it has been reported to be significantly upregulated in uterine cervical cancer tissues [23].

In conclusion, at the present study, several CRC-related diff-miRNAs, diff-lncRNAs, and diff-mRNAs were obtained. A ceRNA network was also constructed to analyze the crosstalk among the identified diff-miRNAs, diff-lncRNAs, and diff-mRNAs. The lncRNA RP11-108 K3.2 and mRNA ONECUT2 in the network may play a crucial role in CRC, with their low expression levels being correlated with better prognosis. Although the mechanism of RP11-108K3.2 and ONECUT2 remains to be revealed, both of the two may be used as novel clinical predictors of CRC. Further experimental studies are needed to reveal the mechanism of RP11-108K3.2 and ONECUT2 in CRC in the future.

## Figures and Tables

**Table 1:** KEGG enrichment pathway.

	KEGG pathway	Count	*p* value
Upregulated	hsa04110: cell cycle	15	1.09*E*-09
	hsa04114: oocyte meiosis	10	1.98*E*-05
	hsa04914: progesterone-mediated oocyte maturation	9	2.35*E*-05
	hsa04060: cytokine-cytokine receptor interaction	14	2.56*E*-05
	hsa05322: systemic lupus erythematosus	8	2.42*E*-03
	hsa05323: rheumatoid arthritis	6	7.18*E*-03
	hsa05034: alcoholism	8	1.09*E*-02
	hsa04062: chemokine signaling pathway	8	1.41*E*-02
	hsa05132: Salmonella infection	5	2.74*E*-02
	hsa03460: Fanconi anemia pathway	4	3.64*E*-02

Downregulated	hsa05410: hypertrophic cardiomyopathy (HCM)	9	7.97*E*-04
	hsa05414: dilated cardiomyopathy	9	1.30*E*-03
	hsa04022: cGMP-PKG signaling pathway	12	2.32*E*-03
	hsa04024: cAMP signaling pathway	13	4.68*E*-03
	hsa04080: neuroactive ligand-receptor interaction	16	4.74*E*-03
	hsa04020: calcium signaling pathway	12	6.04*E*-03
	hsa04724: glutamatergic synapse	9	8.61*E*-03
	hsa04261: adrenergic signaling in cardiomyocytes	10	8.69*E*-03
	hsa04713: circadian entrainment	8	1.06*E*-02
	hsa00980: metabolism of xenobiotics by cytochrome P450	7	1.13*E*-02
	hsa04911: insulin secretion	7	2.13*E*-02
	hsa00071: fatty acid degradation	5	2.18*E*-02
	hsa04725: cholinergic synapse	8	2.33*E*-02
	hsa04726: serotonergic synapse	8	2.33*E*-02
	hsa00830: retinol metabolism	6	2.34*E*-02
	hsa04978: mineral absorption	5	2.54*E*-02
	hsa05412: arrhythmogenic right ventricular cardiomyopathy (ARVC)	6	2.79*E*-02
	hsa03320: PPAR signaling pathway	6	2.79*E*-02
	hsa04920: adipocytokine signaling pathway	6	3.29*E*-02
	hsa04921: oxytocin signaling pathway	9	3.82*E*-02
	hsa04728: dopaminergic synapse	8	4.54*E*-02

**Table 2:** KEGG enrichment pathway of modules.

	KEGG pathway	Count	*p* value
Module-A	hsa04110: cell cycle	12	5.33*E*-17
	hsa04114: oocyte meiosis	9	2.09*E*-11
	hsa04914: progesterone-mediated oocyte maturation	7	1.57*E*-08
	hsa04115: p53 signaling pathway	3	9.04*E*-03

Module-B	hsa04062: chemokine signaling pathway	9	3.79*E*-11
	hsa04060: cytokine-cytokine receptor interaction	7	7.28*E*-07
	hsa05134: legionellosis	3	3.18*E*-03
	hsa04621: NOD-like receptor signaling pathway	3	3.42*E*-03
	hsa05132: Salmonella infection	3	7.37*E*-03
	hsa05323: rheumatoid arthritis	3	8.26*E*-03
	hsa04668: TNF signaling pathway	3	1.20*E*-02
	hsa05200: pathways in cancer	4	2.17*E*-02

**Table 3:** KEGG enrichment pathway of diff-mRNAs in ceRNA network.

KEGG pathway	*p* value	Count
hsa04724: glutamatergic synapse	3.40*E*-03	5
hsa05410: hypertrophic cardiomyopathy (HCM)	6.85*E*-03	4
hsa04725: cholinergic synapse	1.76*E*-02	4
hsa04530: tight junction	1.76*E*-02	5
hsa04726: serotonergic synapse	1.92*E*-02	4
hsa05230: central carbon metabolism in cancer	2.01*E*-02	3
hsa04360: axon guidance	2.24*E*-02	5
hsa04920: adipocytokine signaling pathway	2.35*E*-02	3
hsa05412: arrhythmogenic right ventricular cardiomyopathy (ARVC)	2.62*E*-02	3
hsa04728: dopaminergic synapse	2.93*E*-02	4
hsa00980: metabolism of xenobiotics by cytochrome P450	3.01*E*-02	3
hsa04514: cell adhesion molecules (CAMs)	3.94*E*-02	4
hsa05032: morphine addiction	4.74*E*-02	3
hsa05414: dilated cardiomyopathy (DCM)	4.74*E*-02	3
hsa05033: nicotine addiction	4.93*E*-02	2

## Data Availability

Answer: Yes. Comment: The datasets generated during the current study are not publicly available but identified and anonymized information is potentially available on reasonable request.

## Additional Points

*Highlights*. (1) A total of 688, 241, and 103 differentially expressed mRNA (diff-mRNA), diff-lncRNA, and diff-miRNA were obtained. (2) In the ceRNA network, a total of 315 edges were obtained. (3) lncRNA RP11-108K3.2 and mRNA ONECUT2 correlated with prognosis

## Supplementary Materials

Supplementary Materials Supplementary table 1: the differentially expressed lncRNAs (diff-lncRNAs). Supplementary table 2: the differentially expressed miRNAs (diff-miRNAs). Supplementary table 3: the differentially expressed mRNAs (diff-mRNAs). Supplementary table 4: the differentially expressed miRNAs (diff-miRNAs).
