# Supplementary material for: Construction of ceRNA Coexpression Network and Screening of Molecular Targets in Colorectal Cancer
Source: Dis Markers. 2020 Apr 21;2020:2860582. doi: 10.1155/2020/2860582 (PMC7191371; doi:10.1155/2020/2860582)
Supplement: Supplementary Materials — Supplementary table 1: the differentially expressed lncRNAs (diff-lncRNAs). Supplementary table 2: the differentially expressed miRNAs (diff-miRNAs). Supplementary table 3: the differentially expressed mRNAs (diff-mRNAs). Supplementary table 4: the differentially expressed miRNAs (diff-miRNAs). [file 2860582.f1.docx]

Supplementary table 1 The differentially expressed lncRNAs (diff-lncRNAs)

| lncRNA | logFC | AveExpr | t | P.Value | adj.P.Val | B |
| --- | --- | --- | --- | --- | --- | --- |
| LOC401585 | 5.304836 | 5.177077 | 18.61350952 | 4.02E-14 | 4.94E-10 | 21.89144 |
| RP11-132A1.4 | 3.636063 | 6.463672 | 14.27067502 | 5.77E-12 | 1.58E-08 | 17.4183 |
| RP11-710E1.2 | -3.67593 | 4.156928 | -13.45511728 | 1.69E-11 | 3.46E-08 | 16.41592 |
| CH17-360D5.2 | -2.43006 | 8.312507 | -12.50481616 | 6.31E-11 | 6.75E-08 | 15.16995 |
| SNORA69 | 2.507485 | 3.384822 | 12.19575895 | 9.86E-11 | 8.99E-08 | 14.74542 |
| VPS9D1-AS1 | 2.531052 | 8.078571 | 11.83836348 | 1.67E-10 | 1.33E-07 | 14.24206 |
| RP1-283K11.2 | -2.16749 | 3.229963 | -11.65180246 | 2.21E-10 | 1.55E-07 | 13.97386 |
| RP11-728F11.4 | -2.34621 | 6.143871 | -10.5979612 | 1.15E-09 | 4.28E-07 | 12.38526 |
| RP13-514E23.1 | -2.18445 | 6.172639 | -10.50387109 | 1.34E-09 | 4.84E-07 | 12.23709 |
| MIR497HG | -2.12383 | 6.757206 | -9.957922049 | 3.31E-09 | 8.65E-07 | 11.35586 |
| PGM5-AS1 | -6.29083 | 8.003317 | -9.902384211 | 3.63E-09 | 9.41E-07 | 11.26412 |
| AC002398.12 | -5.55839 | 6.393034 | -9.48923941 | 7.40E-09 | 1.57E-06 | 10.56919 |
| LINC01354 | -2.65036 | 5.154377 | -9.378797714 | 8.98E-09 | 1.79E-06 | 10.37965 |
| MIR30C2 | -2.06482 | 7.22088 | -9.369484012 | 9.13E-09 | 1.80E-06 | 10.36359 |
| CCAT1 | 5.77126 | 7.466308 | 9.313385175 | 1.01E-08 | 1.92E-06 | 10.26662 |
| RP11-483C6.1 | -2.52444 | 3.302516 | -9.299587267 | 1.03E-08 | 1.92E-06 | 10.24271 |
| PGM5P2 | -4.48495 | 6.504469 | -9.289060454 | 1.05E-08 | 1.94E-06 | 10.22444 |
| AC116035.1 | -2.6946 | 3.862142 | -9.001261276 | 1.76E-08 | 2.81E-06 | 9.719408 |
| PGM5P3-AS1 | -2.22355 | 5.854464 | -8.968753067 | 1.87E-08 | 2.92E-06 | 9.661658 |
| DPY19L2P4 | -2.42703 | 5.195653 | -8.916632364 | 2.05E-08 | 3.15E-06 | 9.568767 |
| RIMKLB | -3.02637 | 9.93396 | -8.853726668 | 2.30E-08 | 3.35E-06 | 9.45616 |
| AOC4P | -2.73369 | 6.522699 | -8.625809145 | 3.50E-08 | 4.50E-06 | 9.043616 |
| RBMS3-AS3 | -3.19491 | 5.639801 | -8.564802965 | 3.92E-08 | 4.77E-06 | 8.931973 |
| LOC101928134 | -4.60583 | 5.267208 | -8.504244062 | 4.38E-08 | 5.09E-06 | 8.820637 |
| NBEAP1 | -2.74644 | 5.203476 | -8.39657504 | 5.37E-08 | 5.59E-06 | 8.621428 |
| LINC00659 | 2.438633 | 7.222838 | 8.282621742 | 6.65E-08 | 6.52E-06 | 8.408825 |
| RP3-331H24.6 | -2.49583 | 7.997954 | -8.063764769 | 1.01E-07 | 9.05E-06 | 7.995381 |
| CASC19 | 3.394338 | 4.902922 | 8.049982729 | 1.04E-07 | 9.22E-06 | 7.96912 |
| RP11-286H15.1 | -3.97522 | 9.195169 | -7.996863112 | 1.15E-07 | 9.76E-06 | 7.867648 |
| RP11-49G2.3 | -2.59789 | 3.666284 | -7.889818946 | 1.42E-07 | 1.12E-05 | 7.661955 |
| MIR145 | -4.59619 | 5.017405 | -7.80351736 | 1.68E-07 | 1.28E-05 | 7.494939 |
| RP11-135D11.2 | -3.04071 | 4.331285 | -7.78428776 | 1.74E-07 | 1.30E-05 | 7.45758 |
| HMGB3P1 | 2.420542 | 8.489193 | 7.723623418 | 1.96E-07 | 1.44E-05 | 7.339381 |
| LINC00702 | -2.33152 | 7.944419 | -7.564747545 | 2.69E-07 | 1.76E-05 | 7.027353 |
| CADM3-AS1 | -2.73568 | 5.044799 | -7.53789992 | 2.84E-07 | 1.82E-05 | 6.974271 |
| RP11-57A1.1 | 2.217052 | 4.475814 | 7.437983395 | 3.47E-07 | 2.05E-05 | 6.775826 |
| CLEC4GP1 | -2.42924 | 4.372631 | -7.408524738 | 3.68E-07 | 2.15E-05 | 6.717048 |
| AC123023.1 | 4.738569 | 4.717877 | 7.378824596 | 3.90E-07 | 2.24E-05 | 6.657665 |
| RP11-6O2.3 | -4.8697 | 6.228947 | -7.324614884 | 4.36E-07 | 2.39E-05 | 6.548955 |
| CNTNAP3P2 | -3.06398 | 5.251095 | -7.316440374 | 4.43E-07 | 2.42E-05 | 6.532526 |
| CTD-2008A1.3 | 3.540254 | 5.163167 | 7.285859231 | 4.71E-07 | 2.51E-05 | 6.470982 |
| MIR143HG | -2.77132 | 7.659936 | -7.255821964 | 5.01E-07 | 2.64E-05 | 6.410405 |
| BRCAT54 | -3.44988 | 4.920668 | -7.245771554 | 5.11E-07 | 2.67E-05 | 6.390107 |
| DIRC3 | -2.36156 | 4.217901 | -7.1106665 | 6.74E-07 | 3.23E-05 | 6.115879 |
| ADAMTS9-AS1 | -3.46814 | 5.809299 | -7.0938578 | 6.98E-07 | 3.31E-05 | 6.081583 |
| CP | -3.4737 | 5.367155 | -7.087032751 | 7.08E-07 | 3.34E-05 | 6.067646 |
| AF001548.4 | -4.93739 | 8.682597 | -7.079621994 | 7.19E-07 | 3.38E-05 | 6.052506 |
| HAND2-AS1 | -3.33582 | 5.159008 | -7.063873835 | 7.42E-07 | 3.45E-05 | 6.020307 |
| RP11-307B6.3 | -3.31446 | 6.131195 | -7.050614857 | 7.63E-07 | 3.48E-05 | 5.993171 |
| RP11-435O5.2 | 2.681385 | 4.045433 | 6.983475022 | 8.76E-07 | 3.80E-05 | 5.855385 |
| LINC00114 | 3.331936 | 5.388559 | 6.961960254 | 9.16E-07 | 3.92E-05 | 5.8111 |
| CTD-2619J13.17 | -2.32033 | 5.36532 | -6.883138388 | 1.08E-06 | 4.34E-05 | 5.648311 |
| RP11-342A23.2 | -2.2642 | 6.066477 | -6.833901664 | 1.20E-06 | 4.67E-05 | 5.54619 |
| ZNF542P | -2.33467 | 4.044822 | -6.79374539 | 1.30E-06 | 4.89E-05 | 5.462657 |
| AF186192.1 | -2.32782 | 5.82061 | -6.67317563 | 1.68E-06 | 5.80E-05 | 5.210533 |
| FAM83C-AS1 | 2.142823 | 4.972272 | 6.631722003 | 1.83E-06 | 6.20E-05 | 5.123396 |
| LINC00853 | 2.063534 | 5.276592 | 6.624325827 | 1.86E-06 | 6.25E-05 | 5.107825 |
| RP11-148L24.1 | -2.5958 | 5.732483 | -6.605978375 | 1.93E-06 | 6.41E-05 | 5.069166 |
| RP11-1149O23.4 | 2.208158 | 4.166553 | 6.597528876 | 1.97E-06 | 6.48E-05 | 5.051348 |
| RP11-305O6.3 | -2.87953 | 6.372853 | -6.584067409 | 2.03E-06 | 6.64E-05 | 5.022941 |
| RP1-146A15.1 | -3.16271 | 5.451377 | -6.563238889 | 2.12E-06 | 6.88E-05 | 4.97894 |
| RP11-203J24.9 | -2.19397 | 9.906211 | -6.501240179 | 2.42E-06 | 7.55E-05 | 4.847626 |
| LOC283856 | -2.07967 | 3.781594 | -6.474752099 | 2.56E-06 | 7.91E-05 | 4.791369 |
| LOC101928796 | -2.27602 | 5.610903 | -6.45460534 | 2.67E-06 | 8.15E-05 | 4.748519 |
| LOC399815 | 2.414305 | 4.001704 | 6.452466103 | 2.68E-06 | 8.17E-05 | 4.743966 |
| MIR4697HG | -3.2708 | 7.927596 | -6.425638326 | 2.84E-06 | 8.51E-05 | 4.686817 |
| RP13-753N3.3 | 2.047682 | 7.750184 | 6.39670319 | 3.02E-06 | 8.82E-05 | 4.625074 |
| MAGI2-AS3 | -2.10118 | 6.990964 | -6.391471791 | 3.06E-06 | 8.87E-05 | 4.6139 |
| RP11-318A15.8 | -2.344 | 4.663667 | -6.388159129 | 3.08E-06 | 8.90E-05 | 4.606822 |
| KGFLP2 | -2.29624 | 8.129831 | -6.375413949 | 3.17E-06 | 9.07E-05 | 4.579577 |
| LOC100507073 | -2.8033 | 6.557286 | -6.348002977 | 3.36E-06 | 9.46E-05 | 4.520912 |
| LINC01279 | -2.71813 | 11.96702 | -6.333594054 | 3.46E-06 | 9.69E-05 | 4.490035 |
| LINC00460 | 3.345498 | 4.633085 | 6.326606165 | 3.52E-06 | 9.78E-05 | 4.475052 |
| LINC00920 | 2.727637 | 5.774646 | 6.30286784 | 3.70E-06 | 0.000101 | 4.424104 |
| LA16c-329F2.2 | 2.363041 | 5.124788 | 6.279656529 | 3.89E-06 | 0.000104 | 4.374219 |
| MIR27B | -2.40718 | 6.232398 | -6.232451411 | 4.31E-06 | 0.000113 | 4.272556 |
| RP3-422G23.4 | -4.69079 | 4.626161 | -6.231127786 | 4.32E-06 | 0.000113 | 4.269702 |
| CTD-2033D15.3 | -2.91805 | 6.143388 | -6.191863151 | 4.71E-06 | 0.00012 | 4.184922 |
| SNORA35 | -2.04534 | 5.401859 | -6.176120255 | 4.87E-06 | 0.000124 | 4.150876 |
| AF001548.3 | -4.29581 | 7.248354 | -6.170060184 | 4.94E-06 | 0.000125 | 4.137762 |
| RP11-783K16.5 | 2.47882 | 8.166843 | 6.159780203 | 5.05E-06 | 0.000126 | 4.115506 |
| FOXD3-AS1 | -3.26093 | 7.035131 | -6.08508217 | 5.94E-06 | 0.00014 | 3.9534 |
| AC116614.1 | -2.68142 | 6.213753 | -6.081662659 | 5.99E-06 | 0.000141 | 3.945963 |
| NAV2-IT1 | -2.04801 | 4.179585 | -6.081621083 | 5.99E-06 | 0.000141 | 3.945872 |
| AC005498.3 | -2.84869 | 4.64656 | -6.073589391 | 6.10E-06 | 0.000143 | 3.928398 |
| AP000892.6 | -3.83698 | 13.04481 | -6.070593957 | 6.14E-06 | 0.000143 | 3.92188 |
| TRHDE-AS1 | -3.22435 | 3.620135 | -6.05919254 | 6.29E-06 | 0.000146 | 3.897057 |
| XXbac-BPG13B8.10 | -2.69315 | 4.41855 | -6.030450518 | 6.70E-06 | 0.000153 | 3.834413 |
| RP11-350J20.12 | 2.559868 | 7.381473 | 6.030298213 | 6.70E-06 | 0.000153 | 3.834081 |
| AP001627.1 | -2.16351 | 3.831944 | -5.990472194 | 7.32E-06 | 0.000163 | 3.747116 |
| ZIM2-AS1 | -2.17094 | 3.753881 | -5.955742782 | 7.90E-06 | 0.000172 | 3.671128 |
| LOC101060019 | -3.0259 | 3.495921 | -5.931396593 | 8.33E-06 | 0.00018 | 3.617775 |
| THRB-IT1 | -2.447 | 9.861381 | -5.881324168 | 9.31E-06 | 0.000195 | 3.50783 |
| RP11-999E24.3 | -2.12215 | 7.590203 | -5.869506857 | 9.55E-06 | 0.0002 | 3.481841 |
| RP11-706O15.3 | 2.077279 | 9.794002 | 5.86638736 | 9.62E-06 | 0.0002 | 3.474978 |
| SNORD115-30 | -2.8868 | 4.079704 | -5.858212177 | 9.80E-06 | 0.000203 | 3.456986 |
| RP11-73M7.1 | 2.642996 | 4.132037 | 5.832176292 | 1.04E-05 | 0.000213 | 3.399638 |
| CTD-2298J14.2 | -2.56312 | 4.308419 | -5.795957456 | 1.12E-05 | 0.000227 | 3.319735 |
| SNORD115-2 | -3.32901 | 4.70616 | -5.785713876 | 1.15E-05 | 0.000231 | 3.29711 |
| SNORD115-22 | -3.42302 | 4.78252 | -5.777859748 | 1.17E-05 | 0.000235 | 3.279755 |
| KGFLP1 | -2.03091 | 7.594583 | -5.77536807 | 1.18E-05 | 0.000236 | 3.274248 |
| SNORD116-9 | -2.0971 | 11.01793 | -5.761399087 | 1.21E-05 | 0.000241 | 3.24336 |
| RP11-701H24.7 | -2.40661 | 5.82785 | -5.760301184 | 1.22E-05 | 0.000242 | 3.240932 |
| RP11-753H16.3 | -4.5551 | 4.694137 | -5.733062169 | 1.29E-05 | 0.000254 | 3.180638 |
| SVILP1 | -2.40165 | 9.472696 | -5.725253796 | 1.32E-05 | 0.000257 | 3.163339 |
| RP11-389G6.3 | -5.10687 | 4.799593 | -5.722214769 | 1.33E-05 | 0.000258 | 3.156605 |
| RP11-506O24.2 | -2.43926 | 5.315488 | -5.718137386 | 1.34E-05 | 0.000259 | 3.147568 |
| RP11-720L2.3 | -2.53283 | 7.432828 | -5.714946059 | 1.35E-05 | 0.00026 | 3.140494 |
| RP11-266K4.13 | -2.20419 | 4.444872 | -5.712062108 | 1.36E-05 | 0.000262 | 3.1341 |
| MIR938 | -2.37138 | 5.587504 | -5.70116512 | 1.39E-05 | 0.000266 | 3.109933 |
| LOC101929596 | -2.6925 | 5.81725 | -5.691356642 | 1.42E-05 | 0.00027 | 3.088169 |
| CTD-2349P21.11 | 2.169196 | 5.134936 | 5.688120264 | 1.43E-05 | 0.000272 | 3.080985 |
| RP11-164N3.2 | -2.00825 | 7.160857 | -5.632673673 | 1.62E-05 | 0.000296 | 2.957747 |
| SNORD116-5 | -2.05363 | 10.41525 | -5.605370904 | 1.72E-05 | 0.00031 | 2.896946 |
| SNORD115-15 | -3.29229 | 5.192961 | -5.594224809 | 1.77E-05 | 0.000315 | 2.872103 |
| RACGAP1P | 2.007105 | 5.550697 | 5.582044752 | 1.81E-05 | 0.00032 | 2.844941 |
| RP11-568A7.2 | 2.511935 | 3.866911 | 5.554020246 | 1.93E-05 | 0.000337 | 2.782389 |
| FEZF1-AS1 | 3.259117 | 6.182908 | 5.551367344 | 1.94E-05 | 0.000338 | 2.776463 |
| LOC101928489 | -2.36717 | 5.595306 | -5.549894274 | 1.95E-05 | 0.000338 | 2.773173 |
| UG0898H09 | -3.42458 | 4.877282 | -5.548777808 | 1.95E-05 | 0.000339 | 2.770679 |
| PWAR4 | -2.57908 | 4.420295 | -5.543161854 | 1.98E-05 | 0.000342 | 2.758131 |
| RP11-451L19.1 | -2.07838 | 3.279412 | -5.524976646 | 2.06E-05 | 0.000353 | 2.71748 |
| AC016735.1 | 2.306936 | 6.694759 | 5.501208739 | 2.18E-05 | 0.000366 | 2.664301 |
| SNORD116-24 | -2.05153 | 12.93129 | -5.46794471 | 2.35E-05 | 0.000388 | 2.589783 |
| SNORD116-4 | -2.02274 | 10.21953 | -5.464501112 | 2.36E-05 | 0.000389 | 2.582063 |
| RP11-650L12.2 | -2.67679 | 5.142141 | -5.45697176 | 2.40E-05 | 0.000393 | 2.565179 |
| C20orf166-AS1 | -2.02804 | 5.35607 | -5.455415362 | 2.41E-05 | 0.000394 | 2.561688 |
| SNORD115-44 | -3.14463 | 5.052338 | -5.454552876 | 2.42E-05 | 0.000395 | 2.559753 |
| LL22NC03-N64E9.1 | 2.31882 | 6.486918 | 5.45336446 | 2.42E-05 | 0.000395 | 2.557088 |
| SNORD116-15 | -2.03438 | 12.99908 | -5.448894387 | 2.45E-05 | 0.000398 | 2.54706 |
| SNORD115-8 | -3.26195 | 4.672015 | -5.43904519 | 2.50E-05 | 0.000404 | 2.524958 |
| AC068137.1 | -3.10094 | 9.554646 | -5.418609122 | 2.62E-05 | 0.000417 | 2.479071 |
| LINC00239 | 2.945064 | 7.220367 | 5.401847213 | 2.72E-05 | 0.000428 | 2.441406 |
| CRNDE | 2.566013 | 6.380136 | 5.401211005 | 2.73E-05 | 0.000428 | 2.439976 |
| CTB-167B5.2 | -2.3248 | 5.803674 | -5.379426096 | 2.87E-05 | 0.000445 | 2.390984 |
| SNORD116-22 | -2.03265 | 13.06909 | -5.37934311 | 2.87E-05 | 0.000445 | 2.390797 |
| SNORD116-6 | -2.05402 | 11.34594 | -5.364804226 | 2.96E-05 | 0.000455 | 2.358077 |
| RP11-418I22.2 | -2.02345 | 7.877679 | -5.364063308 | 2.97E-05 | 0.000455 | 2.356409 |
| RP11-396O20.2 | -2.97638 | 4.378037 | -5.336153182 | 3.16E-05 | 0.000479 | 2.293543 |
| RP11-680F20.12 | -2.29128 | 4.331338 | -5.325541348 | 3.24E-05 | 0.000487 | 2.269622 |
| SNORD116-14 | -2.00782 | 12.8269 | -5.308464935 | 3.37E-05 | 0.000501 | 2.23111 |
| RP11-158I9.5 | -4.09905 | 5.404007 | -5.273866065 | 3.64E-05 | 0.00053 | 2.153003 |
| SNAR-E | 3.411907 | 14.65059 | 5.265067425 | 3.72E-05 | 0.000537 | 2.133124 |
| LOC101928812 | -2.83151 | 5.358546 | -5.24701617 | 3.87E-05 | 0.000556 | 2.092322 |
| SNORD115-20 | -2.92261 | 4.899612 | -5.235443995 | 3.98E-05 | 0.000567 | 2.06615 |
| RP1-193H18.3 | -3.33925 | 3.616303 | -5.232478643 | 4.00E-05 | 0.000569 | 2.059442 |
| RP11-350N15.3 | -2.62616 | 3.955752 | -5.220165303 | 4.12E-05 | 0.000581 | 2.03158 |
| LOC101926964 | 2.440609 | 4.933714 | 5.216714866 | 4.15E-05 | 0.000583 | 2.02377 |
| SNAR-C3 | 3.278397 | 14.60317 | 5.213202225 | 4.18E-05 | 0.000586 | 2.015819 |
| AP001347.6 | -2.05849 | 10.0881 | -5.207670815 | 4.24E-05 | 0.000591 | 2.003295 |
| RP11-550I24.2 | -2.04992 | 4.045139 | -5.186996571 | 4.44E-05 | 0.000613 | 1.956467 |
| RP11-392A14.8 | -2.07304 | 5.437284 | -5.184768888 | 4.46E-05 | 0.000616 | 1.95142 |
| SNORD115-41 | -3.08135 | 5.29254 | -5.175055049 | 4.56E-05 | 0.000628 | 1.929404 |
| UCA1 | 3.857169 | 9.949428 | 5.161150356 | 4.71E-05 | 0.00064 | 1.897879 |
| SNAR-C1 | 3.41316 | 14.11994 | 5.152201072 | 4.81E-05 | 0.000651 | 1.87758 |
| LOC101928731 | -3.38983 | 5.310648 | -5.134155549 | 5.01E-05 | 0.000672 | 1.836633 |
| RP11-338N10.3 | 2.345273 | 5.57266 | 5.045044361 | 6.15E-05 | 0.000783 | 1.634089 |
| CASC21 | 2.66958 | 8.443591 | 5.032132377 | 6.34E-05 | 0.000802 | 1.604696 |
| FOXP2 | -2.42631 | 6.920119 | -5.017099564 | 6.56E-05 | 0.000823 | 1.570462 |
| RNU2-34P | -2.58554 | 3.643687 | -4.980885201 | 7.13E-05 | 0.000878 | 1.487932 |
| RP11-380P13.1 | -2.39305 | 3.514401 | -4.952013949 | 7.62E-05 | 0.00092 | 1.422079 |
| LA16c-60D12.2 | 2.064296 | 5.577956 | 4.929106663 | 8.03E-05 | 0.000959 | 1.369794 |
| CTA-246H3.12 | 3.050129 | 6.210143 | 4.926780163 | 8.08E-05 | 0.000961 | 1.364482 |
| RP11-383J24.1 | 2.785195 | 4.03263 | 4.888318892 | 8.83E-05 | 0.001031 | 1.276625 |
| LINC01154 | -2.59074 | 6.115339 | -4.88399463 | 8.91E-05 | 0.00104 | 1.266742 |
| CTD-2116N20.1 | 2.13329 | 3.732602 | 4.865366402 | 9.31E-05 | 0.001076 | 1.224157 |
| RP11-679B19.1 | -2.00033 | 4.813913 | -4.863869114 | 9.34E-05 | 0.001078 | 1.220733 |
| RP11-932O9.10 | 2.410906 | 6.491184 | 4.863728279 | 9.34E-05 | 0.001078 | 1.220411 |
| AC104946.1 | -3.02733 | 8.779339 | -4.847018567 | 9.71E-05 | 0.00111 | 1.182196 |
| LINC00473 | -2.04974 | 5.038918 | -4.822530507 | 0.000103 | 0.001163 | 1.126166 |
| ARHGEF38-IT1 | 2.158472 | 5.572341 | 4.816412647 | 0.000104 | 0.001174 | 1.112163 |
| RP11-554A11.4 | -2.46209 | 7.375394 | -4.815392572 | 0.000104 | 0.001176 | 1.109828 |
| AC132825.1 | -2.92286 | 10.16355 | -4.813006762 | 0.000105 | 0.00118 | 1.104367 |
| RP11-475B2.1 | -2.73479 | 3.99343 | -4.796098703 | 0.000109 | 0.001215 | 1.065656 |
| LOC100506725 | -2.22044 | 3.295091 | -4.732680961 | 0.000127 | 0.001357 | 0.920353 |
| SNAR-H | 3.474125 | 13.4547 | 4.711709861 | 0.000133 | 0.001407 | 0.872268 |
| RP11-243M5.4 | -2.60396 | 4.729695 | -4.663725988 | 0.000149 | 0.001523 | 0.762187 |
| RP11-532F6.3 | -2.15017 | 4.61419 | -4.619951388 | 0.000164 | 0.001648 | 0.661699 |
| SNAR-A14 | 2.076075 | 15.95649 | 4.594912991 | 0.000174 | 0.001727 | 0.604197 |
| RP11-1334A24.5 | -2.12362 | 5.044275 | -4.553151068 | 0.000192 | 0.001866 | 0.508255 |
| LOC283683 | -2.31572 | 3.418647 | -4.533801601 | 0.000201 | 0.001929 | 0.463791 |
| SNAR-A13 | 2.053966 | 15.95382 | 4.51585539 | 0.00021 | 0.001994 | 0.422545 |
| MIR5690 | -2.05688 | 5.498487 | -4.48031078 | 0.000228 | 0.002106 | 0.340838 |
| LOC441666 | 2.284708 | 6.364747 | 4.437336192 | 0.000252 | 0.002267 | 0.242033 |
| RP11-297C4.7 | -2.2752 | 6.820744 | -4.426849949 | 0.000258 | 0.00231 | 0.217922 |
| SERTAD4-AS1 | -2.47259 | 10.6569 | -4.422947901 | 0.00026 | 0.002323 | 0.208949 |
| RP11-20G13.3 | 2.308012 | 3.894924 | 4.393954582 | 0.000279 | 0.00245 | 0.14228 |
| RP11-2N1.3 | -2.18002 | 5.080877 | -4.39198659 | 0.00028 | 0.002459 | 0.137755 |
| RP11-138J23.1 | 2.948147 | 3.439557 | 4.386955224 | 0.000283 | 0.002484 | 0.126185 |
| RP11-546K22.1 | 2.704141 | 3.484397 | 4.361182034 | 0.000301 | 0.002607 | 0.06692 |
| RP11-150O12.3 | 2.815984 | 7.923236 | 4.349852665 | 0.000309 | 0.002661 | 0.040869 |
| AC007392.3 | -2.40807 | 3.690252 | -4.344105707 | 0.000313 | 0.002689 | 0.027655 |
| MGC32805 | 2.530609 | 3.874727 | 4.30263476 | 0.000345 | 0.002888 | -0.0677 |
| RP11-79H23.3 | 2.037217 | 5.368108 | 4.260085339 | 0.000381 | 0.003118 | -0.16551 |
| RP11-108K3.2 | 2.908223 | 5.591262 | 4.240781598 | 0.000399 | 0.003234 | -0.20988 |
| AC074011.2 | -2.0081 | 4.936484 | -4.177679162 | 0.000462 | 0.003637 | -0.35485 |
| LOC101927969 | -2.54147 | 4.279932 | -4.131431401 | 0.000515 | 0.003936 | -0.46104 |
| RP11-734K21.5 | 2.54965 | 4.176896 | 4.088083777 | 0.00057 | 0.004244 | -0.5605 |
| RP11-317L10.1 | -2.10209 | 3.483929 | -4.079733762 | 0.000581 | 0.004313 | -0.57965 |
| LINC00304 | -2.47975 | 4.450951 | -4.071914695 | 0.000592 | 0.004369 | -0.59758 |
| AC008269.2 | -2.45054 | 4.286676 | -4.034972015 | 0.000645 | 0.004669 | -0.68226 |
| RP11-284F21.10 | 2.47778 | 10.37058 | 3.996921642 | 0.000706 | 0.004997 | -0.76941 |
| RP11-545G3.1 | -2.41997 | 5.159426 | -3.996243601 | 0.000707 | 0.005003 | -0.77096 |
| RP11-15F12.1 | 2.045028 | 5.660325 | 3.977236565 | 0.000739 | 0.005183 | -0.81446 |
| AL450304.1 | 2.085341 | 11.21268 | 3.94057297 | 0.000805 | 0.005538 | -0.89831 |
| AC012667.1 | 2.042548 | 12.71228 | 3.914218761 | 0.000856 | 0.005816 | -0.95854 |
| RP11-53M11.3 | 2.277453 | 3.909215 | 3.889497567 | 0.000907 | 0.00609 | -1.01498 |
| LINC00970 | 2.239641 | 3.479323 | 3.875134106 | 0.000938 | 0.00626 | -1.04776 |
| MKX-AS1 | -2.26191 | 3.802948 | -3.855015137 | 0.000983 | 0.006472 | -1.09365 |
| AL590726.1 | 2.030186 | 13.70374 | 3.781697998 | 0.001167 | 0.007392 | -1.26058 |
| PCAT7 | 2.135224 | 5.796954 | 3.751783303 | 0.001251 | 0.007783 | -1.32855 |
| LINC01556 | 2.22825 | 3.879803 | 3.740423341 | 0.001285 | 0.00795 | -1.35435 |
| LINC01260 | 2.318903 | 3.793599 | 3.73906688 | 0.001289 | 0.007971 | -1.35742 |
| POU6F2-AS1 | 3.205384 | 4.193447 | 3.737612741 | 0.001294 | 0.007988 | -1.36072 |
| SNORD115-35 | -2.43501 | 4.927854 | -3.737300845 | 0.001294 | 0.00799 | -1.36143 |
| EVADR | 3.717395 | 7.588292 | 3.734652453 | 0.001303 | 0.008031 | -1.36744 |
| RP11-511B23.2 | 2.145379 | 3.9453 | 3.728711032 | 0.001321 | 0.008107 | -1.38092 |
| AC079005.1 | 2.00611 | 12.99384 | 3.720920502 | 0.001345 | 0.008229 | -1.39859 |
| DPP10-AS1 | -2.08919 | 4.598214 | -3.680597405 | 0.001477 | 0.008859 | -1.48995 |
| RP11-1069G10.1 | -2.33864 | 6.626947 | -3.670679495 | 0.001512 | 0.009029 | -1.5124 |
| RP11-364P22.3 | 2.016693 | 2.956043 | 3.639340584 | 0.001626 | 0.009559 | -1.58324 |
| CH17-351M24.1 | -2.22153 | 4.271628 | -3.586126818 | 0.001841 | 0.010507 | -1.70327 |
| B3GALT5-AS1 | -3.16584 | 6.668029 | -3.584595254 | 0.001847 | 0.010535 | -1.70672 |
| AL163953.2 | 2.509231 | 3.327989 | 3.566489544 | 0.001927 | 0.010899 | -1.74748 |
| RP5-1056H1.2 | 2.032679 | 7.493429 | 3.558893093 | 0.001961 | 0.011053 | -1.76456 |
| RP11-474D1.3 | 2.946803 | 4.685167 | 3.48043527 | 0.002352 | 0.012672 | -1.94056 |
| WASIR2 | 2.041 | 3.234345 | 3.37049133 | 0.003033 | 0.015364 | -2.18562 |
| AC006019.3 | -2.01592 | 4.993097 | -3.33816675 | 0.003267 | 0.016265 | -2.25728 |
| CTD-2147F2.1 | 2.076851 | 3.067293 | 3.33151266 | 0.003318 | 0.016455 | -2.27201 |
| RP11-309M23.1 | -2.74951 | 4.670919 | -3.296486457 | 0.003596 | 0.017483 | -2.3494 |
| RP11-575A19.2 | -2.82552 | 5.405349 | -3.295455862 | 0.003604 | 0.017503 | -2.35167 |
| CD36 | -2.63526 | 7.128899 | -3.290702971 | 0.003644 | 0.01765 | -2.36216 |
| AC093732.1 | 2.123443 | 4.000642 | 3.285852944 | 0.003685 | 0.017824 | -2.37285 |
| RP11-763F8.1 | 2.054735 | 3.054024 | 3.251722598 | 0.003985 | 0.018921 | -2.44797 |
| SNORD115-16 | -2.54826 | 5.507189 | -3.198413703 | 0.004502 | 0.020688 | -2.56482 |
| RP11-143E21.3 | 2.779015 | 3.385939 | 3.180713748 | 0.004687 | 0.021368 | -2.60349 |
| RP11-864N7.4 | -2.72931 | 5.911385 | -3.171583822 | 0.004786 | 0.02171 | -2.6234 |
| RP11-465N4.4 | 2.03225 | 8.09701 | 3.087775204 | 0.005791 | 0.025089 | -2.80536 |
| RP11-692C24.1 | 3.146458 | 3.824672 | 2.855194312 | 0.009766 | 0.037369 | -3.30095 |
| RP11-115D19.3 | 2.109719 | 3.099591 | 2.845153873 | 0.009987 | 0.037983 | -3.32199 |
| LINCR-0002 | 2.025184 | 5.090309 | 2.72126369 | 0.013131 | 0.046732 | -3.5791 |

Supplementary table 2. The differentially expressed miRNAs (diff-miRNAs)

| miRNA | logFC | adj.P.Val | P.Value | t | B |
| --- | --- | --- | --- | --- | --- |
| hsa-miR-31-5p | 9.153176 | 0.000598 | 4.60E-06 | 6.38232 | 4.37305 |
| hsa-miR-224-5p | 8.99901 | 0.000259 | 1.12E-06 | 7.0954 | 5.73669 |
| hsa-miR-1244 | 8.326863 | 3.59E-05 | 1.73E-08 | 9.43153 | 9.67758 |
| hsa-miR-188-5p | 7.89375 | 0.000259 | 1.12E-06 | 7.09302 | 5.73227 |
| hsa-miR-764 | 7.667327 | 0.001075 | 1.39E-05 | 5.84593 | 3.29934 |
| hsa-miR-301b | 7.589305 | 0.000667 | 6.73E-06 | 6.19595 | 4.00447 |
| hsa-miR-19b-1-5p | 7.257608 | 0.001277 | 1.84E-05 | 5.71447 | 3.03032 |
| hsa-miR-3648 | 7.016826 | 0.001333 | 2.11E-05 | 5.65045 | 2.89852 |
| hsa-miR-452-3p | 6.820998 | 0.004439 | 1.24E-04 | 4.83587 | 1.18186 |
| hsa-miR-3157-3p | 6.796099 | 0.00085 | 9.40E-06 | 6.03486 | 3.68201 |
| hsa-miR-135b-3p | 6.74844 | 0.000564 | 3.91E-06 | 6.46267 | 4.53042 |
| hsa-miR-412 | 6.676802 | 0.011995 | 5.19E-04 | 4.19415 | -0.20641 |
| hsa-miR-19a-5p | 6.589656 | 0.001333 | 2.03E-05 | 5.66823 | 2.93516 |
| hsa-miR-96-5p | 6.575318 | 0.009219 | 3.16E-04 | 4.41525 | 0.27383 |
| hsa-miR-942 | 6.490987 | 0.001333 | 2.18E-05 | 5.63551 | 2.86769 |
| hsa-miR-18a-3p | 6.44523 | 0.001094 | 1.47E-05 | 5.82034 | 3.24715 |
| hsa-miR-135b-5p | 6.269277 | 0.003636 | 9.14E-05 | 4.97292 | 1.47518 |
| hsa-miR-421 | 6.263953 | 0.01202 | 5.26E-04 | 4.18832 | -0.21906 |
| hsa-miR-545-3p | 6.149096 | 0.010112 | 3.74E-04 | 4.33951 | 0.10944 |
| hsa-miR-34a-3p | 6.101058 | 0.00019 | 2.99E-07 | 7.79195 | 6.99555 |
| hsa-miR-4778-3p | 6.05004 | 0.009263 | 3.29E-04 | 4.39636 | 0.23284 |
| hsa-miR-222-5p | 5.992396 | 0.001099 | 1.53E-05 | 5.80144 | 3.20854 |
| hsa-miR-432-3p | 5.927658 | 0.003636 | 9.26E-05 | 4.9671 | 1.46276 |
| hsa-miR-4524a-5p | 5.91181 | 0.008098 | 2.63E-04 | 4.49761 | 0.45234 |
| hsa-miR-4753-3p | 5.885558 | 0.009219 | 3.21E-04 | 4.40765 | 0.25733 |
| hsa-miR-520e | 5.876938 | 0.009219 | 3.23E-04 | 4.40452 | 0.25054 |
| hsa-miR-3650 | 5.870752 | 0.011148 | 4.40E-04 | 4.26786 | -0.0462 |
| hsa-miR-3605-3p | 5.847395 | 0.011705 | 5.01E-04 | 4.20998 | -0.17198 |
| hsa-miR-625-5p | 5.833786 | 0.012614 | 5.76E-04 | 4.14777 | -0.30722 |
| hsa-miR-31-3p | 5.79268 | 0.032408 | 2.79E-03 | 3.44671 | -1.82297 |
| hsa-miR-3609 | 5.706076 | 0.009886 | 3.56E-04 | 4.36132 | 0.1568 |
| hsa-miR-4295 | 5.695541 | 0.029155 | 2.16E-03 | 3.56154 | -1.57696 |
| hsa-miR-4703-3p | 5.686816 | 0.036374 | 3.34E-03 | 3.36679 | -1.9933 |
| hsa-miR-3127-5p | 5.659287 | 0.01656 | 9.08E-04 | 3.94564 | -0.74652 |
| hsa-miR-374a-3p | 5.633182 | 0.016316 | 8.78E-04 | 3.96055 | -0.71414 |
| hsa-miR-155-3p | 5.6194 | 0.005275 | 1.57E-04 | 4.72791 | 0.94979 |
| hsa-miR-3158-3p | 5.511625 | 0.014754 | 7.44E-04 | 4.03382 | -0.55494 |
| hsa-miR-1226-3p | 5.406087 | 0.008098 | 2.65E-04 | 4.4941 | 0.44473 |
| hsa-miR-573 | 5.358664 | 0.017595 | 1.06E-03 | 3.87846 | -0.89231 |
| hsa-miRPlus-C1114 | 5.316925 | 0.020997 | 1.33E-03 | 3.77583 | -1.11471 |
| hsa-miR-4760-5p | 5.310442 | 0.029295 | 2.23E-03 | 3.54616 | -1.60997 |
| hsa-miR-135a-5p | 5.244314 | 0.047539 | 4.89E-03 | 3.19524 | -2.35585 |
| hsa-miR-3130-5p | 5.221793 | 0.011148 | 4.50E-04 | 4.25772 | -0.06823 |
| hsa-miR-433 | 5.18339 | 0.028336 | 1.97E-03 | 3.60101 | -1.49209 |
| hsa-miR-4326 | 5.171544 | 0.017138 | 9.88E-04 | 3.90817 | -0.82786 |
| hsa-miR-3664-5p | 5.158716 | 0.031663 | 2.68E-03 | 3.46528 | -1.78328 |
| hsa-miR-3920 | 5.129842 | 0.013808 | 6.50E-04 | 4.09381 | -0.42454 |
| hsa-miR-3198 | 5.107234 | 0.021357 | 1.37E-03 | 3.76355 | -1.14129 |
| hsa-miR-519b-3p | 5.072691 | 0.011148 | 4.31E-04 | 4.27634 | -0.02778 |
| hsa-miR-744-3p | 5.06762 | 0.017521 | 1.03E-03 | 3.89105 | -0.86501 |
| hsa-miR-3670 | 5.05351 | 0.037809 | 3.49E-03 | 3.34713 | -2.03506 |
| hsa-miR-3116 | 5.020991 | 0.035091 | 3.14E-03 | 3.39471 | -1.93389 |
| hsa-miR-504 | 4.895788 | 0.042207 | 4.08E-03 | 3.27725 | -2.18309 |
| hsa-miR-450b-5p | 4.879503 | 0.040006 | 3.75E-03 | 3.31488 | -2.10346 |
| hsa-miR-1915-5p | 4.865831 | 0.044535 | 4.39E-03 | 3.24427 | -2.25269 |
| hsa-miR-3913-3p | 4.860894 | 0.018465 | 1.14E-03 | 3.84303 | -0.96914 |
| hsa-miR-4495 | 4.823268 | 0.017595 | 1.06E-03 | 3.8786 | -0.892 |
| hsa-miR-3121-5p | 4.821022 | 0.029295 | 2.25E-03 | 3.5435 | -1.6157 |
| hsa-miR-548d-5p | 4.790041 | 0.024381 | 1.61E-03 | 3.69302 | -1.29374 |
| hsa-miR-296-5p | 4.761064 | 0.048689 | 5.12E-03 | 3.17435 | -2.39966 |
| hsa-miR-4254 | 4.709172 | 0.024528 | 1.63E-03 | 3.68539 | -1.31022 |
| hsa-miR-515-3p | 4.676365 | 0.016169 | 8.62E-04 | 3.96853 | -0.69679 |
| hsa-miR-500a-3p | 4.671667 | 0.028555 | 2.00E-03 | 3.59452 | -1.50605 |
| hsa-miR-4664-5p | 4.655108 | 0.019783 | 1.25E-03 | 3.80561 | -1.05023 |
| hsa-miR-4680-3p | 4.606622 | 0.049609 | 5.41E-03 | 3.1497 | -2.45126 |
| hsa-miR-1263 | 4.566077 | 0.047539 | 4.93E-03 | 3.19135 | -2.364 |
| hsa-miR-105-5p | 4.536957 | 0.03263 | 2.84E-03 | 3.43937 | -1.83865 |
| hsa-miR-5009-5p | 4.512451 | 0.048639 | 5.10E-03 | 3.17688 | -2.39435 |
| hsa-miR-4307 | 4.475916 | 0.040342 | 3.80E-03 | 3.30884 | -2.11627 |
| hsa-miR-4736 | 4.450466 | 0.041667 | 4.00E-03 | 3.28527 | -2.16614 |
| hsa-miR-15a-3p | 4.428238 | 0.021357 | 1.38E-03 | 3.7616 | -1.14549 |
| hsa-miR-760 | 4.313043 | 0.032408 | 2.80E-03 | 3.44489 | -1.82686 |
| hsa-miR-5695 | 4.265596 | 0.028655 | 2.04E-03 | 3.58621 | -1.52392 |
| hsa-miR-3681-5p | 4.235457 | 0.04351 | 4.24E-03 | 3.25915 | -2.22132 |
| hsa-miR-1207-3p | 4.213706 | 0.039846 | 3.71E-03 | 3.31898 | -2.09478 |
| hsa-miR-521 | 4.171604 | 0.028655 | 2.05E-03 | 3.58492 | -1.5267 |
| hsa-miR-5692c | 4.170257 | 0.029747 | 2.39E-03 | 3.51586 | -1.67499 |
| hsa-miR-501-3p | 4.15469 | 0.041017 | 3.90E-03 | 3.29684 | -2.14166 |
| hsa-miR-4766-3p | 4.144989 | 0.029634 | 2.33E-03 | 3.5276 | -1.6498 |
| hsa-miR-32-5p | 4.1374 | 0.048722 | 5.15E-03 | 3.172 | -2.40459 |
| hsa-miR-181a-3p | 4.129411 | 0.047539 | 4.92E-03 | 3.19221 | -2.36219 |
| hsa-miR-4517 | 4.121364 | 0.04473 | 4.47E-03 | 3.23627 | -2.26956 |
| hsa-miR-4647 | 4.091355 | 0.028771 | 2.07E-03 | 3.57914 | -1.53912 |
| hsa-miR-182-5p | 3.955943 | 0.022402 | 1.46E-03 | 3.73385 | -1.20552 |
| hsa-miR-18b-5p | 3.639661 | 0.029295 | 2.24E-03 | 3.54525 | -1.61194 |
| hsa-miR-3165 | 3.406193 | 0.046329 | 4.65E-03 | 3.21781 | -2.30841 |
| hsa-miR-20a-3p | 3.123627 | 0.021902 | 1.42E-03 | 3.74713 | -1.17681 |
| hsa-miR-20b-5p | 2.790386 | 0.011148 | 4.46E-04 | 4.26091 | -0.0613 |
| hsa-miRPlus-A1086 | 2.581994 | 0.002668 | 5.51E-05 | 5.20469 | 1.96749 |
| hsa-miR-203 | 2.465661 | 0.039326 | 3.65E-03 | 3.32718 | -2.0774 |
| hsa-miR-142-3p | 2.319546 | 0.002384 | 4.52E-05 | 5.29568 | 2.15932 |
| hsa-miR-3682-5p | 2.220859 | 0.014754 | 7.29E-04 | 4.04306 | -0.53486 |
| hsa-miR-20a-5p | 2.092364 | 0.018103 | 1.10E-03 | 3.85872 | -0.93512 |
| hsa-miR-657 | 2.074754 | 0.014754 | 7.34E-04 | 4.03988 | -0.54178 |
| hsa-miR-4694-5p | 2.069133 | 0.030816 | 2.59E-03 | 3.47992 | -1.75196 |
| hsa-miR-497-5p | -2.02043 | 0.004967 | 1.43E-04 | -4.76978 | 1.03989 |
| hsa-miR-548at-5p | -2.42233 | 0.000639 | 5.57E-06 | -6.28858 | 4.18828 |
| hsa-miR-3656 | -2.47814 | 0.035191 | 3.18E-03 | -3.38866 | -1.94676 |
| hsa-miR-145-3p | -2.64975 | 0.000642 | 6.17E-06 | -6.23805 | 4.08817 |
| hsa-miR-133b | -3.43307 | 0.00019 | 3.99E-07 | -7.6361 | 6.72019 |
| hsa-miR-133a | -3.47906 | 0.000291 | 1.40E-06 | -6.9795 | 5.52018 |
| hsa-miR-139-5p | -3.5918 | 0.000669 | 7.07E-06 | -6.17235 | 3.95745 |
| hsa-miR-363-3p | -6.57816 | 0.029747 | 2.40E-03 | -3.51384 | -1.67931 |

Supplementary table 3. The differentially expressed mRNAs (diff-mRNAs)

| Symbol | logFC | AveExpr | t | P.Value | adj.P.Val | B |
| --- | --- | --- | --- | --- | --- | --- |
| CEMIP | 3.161677 | 8.379319 | 21.35009 | 2.38E-15 | 2.80E-11 | 24.56944 |
| MAMDC2 | -5.44644 | 9.097906 | -18.0648 | 5.95E-14 | 3.50E-10 | 21.73689 |
| CADM3 | -4.66278 | 8.257922 | -16.7847 | 2.41E-13 | 8.56E-10 | 20.46575 |
| ETV4 | 4.662882 | 6.325334 | 16.61726 | 2.91E-13 | 8.56E-10 | 20.29152 |
| ADCYAP1R1 | -3.1685 | 8.888186 | -15.6851 | 8.64E-13 | 2.03E-09 | 19.28582 |
| PRPH | -5.23651 | 8.864588 | -14.5591 | 3.47E-12 | 6.79E-09 | 17.98387 |
| METTL7A | -2.00816 | 13.31961 | -14.3923 | 4.30E-12 | 7.21E-09 | 17.7823 |
| AFF3 | -2.94274 | 7.577804 | -14.0522 | 6.68E-12 | 9.81E-09 | 17.36407 |
| PRR7 | 3.011451 | 10.12934 | 13.70789 | 1.05E-11 | 1.38E-08 | 16.93043 |
| ADGRB3 | -4.84875 | 4.991446 | -13.394 | 1.61E-11 | 1.89E-08 | 16.52578 |
| PRIMA1 | -5.85642 | 9.712132 | -13.1887 | 2.14E-11 | 2.25E-08 | 16.25632 |
| LYVE1 | -5.36611 | 8.713395 | -13.1351 | 2.30E-11 | 2.25E-08 | 16.18528 |
| ABCA8 | -4.79533 | 9.816637 | -13.0449 | 2.61E-11 | 2.36E-08 | 16.06518 |
| ENC1 | 2.37944 | 10.91028 | 12.89121 | 3.23E-11 | 2.71E-08 | 15.85866 |
| KRT80 | 3.759283 | 6.613016 | 12.83023 | 3.52E-11 | 2.76E-08 | 15.77609 |
| GLP2R | -3.97739 | 6.142611 | -12.7256 | 4.09E-11 | 2.94E-08 | 15.63361 |
| MMP7 | 8.648813 | 9.740778 | 12.69816 | 4.25E-11 | 2.94E-08 | 15.59605 |
| FXYD1 | -3.14762 | 8.358669 | -12.6168 | 4.77E-11 | 3.12E-08 | 15.4843 |
| KLF15 | -2.13211 | 7.346325 | -12.5212 | 5.48E-11 | 3.39E-08 | 15.35203 |
| C16orf89 | -4.34578 | 5.048416 | -12.2736 | 7.84E-11 | 4.61E-08 | 15.0054 |
| SALL4 | 2.509779 | 6.696876 | 12.22054 | 8.48E-11 | 4.74E-08 | 14.93034 |
| FHL1 | -4.36265 | 13.64601 | -12.0557 | 1.08E-10 | 5.77E-08 | 14.69521 |
| CADM2 | -3.96622 | 4.128802 | -11.9831 | 1.20E-10 | 6.15E-08 | 14.59072 |
| FMN2 | -3.23402 | 6.894125 | -11.8698 | 1.43E-10 | 6.70E-08 | 14.42649 |
| PLP1 | -2.86896 | 8.765224 | -11.7315 | 1.76E-10 | 7.94E-08 | 14.22434 |
| TOP1MT | 2.211154 | 5.401397 | 11.67829 | 1.90E-10 | 8.02E-08 | 14.14594 |
| PCSK2 | -4.77356 | 4.549977 | -11.6754 | 1.91E-10 | 8.02E-08 | 14.14173 |
| FABP6 | 3.549184 | 7.476265 | 11.56694 | 2.26E-10 | 9.14E-08 | 13.98095 |
| SYNGR1 | -3.66166 | 7.778489 | -11.432 | 2.78E-10 | 1.09E-07 | 13.77913 |
| KCNB1 | -6.20868 | 6.260278 | -11.3907 | 2.96E-10 | 1.11E-07 | 13.71705 |
| CYP27B1 | 3.005414 | 4.386437 | 11.37754 | 3.02E-10 | 1.11E-07 | 13.69721 |
| SNTG2 | -2.63273 | 3.655038 | -11.3496 | 3.15E-10 | 1.12E-07 | 13.65498 |
| GFRA3 | -3.16643 | 10.37178 | -11.311 | 3.35E-10 | 1.16E-07 | 13.5965 |
| SCN7A | -5.98882 | 7.441248 | -11.2659 | 3.59E-10 | 1.21E-07 | 13.52804 |
| ADH1B | -5.22846 | 6.273907 | -11.2049 | 3.95E-10 | 1.29E-07 | 13.43509 |
| SYT1 | -3.94555 | 8.52595 | -11.0933 | 4.71E-10 | 1.49E-07 | 13.26384 |
| LEMD1 | 5.770365 | 5.571525 | 11.07795 | 4.82E-10 | 1.49E-07 | 13.24016 |
| UST | -2.75633 | 8.160571 | -11.0231 | 5.26E-10 | 1.59E-07 | 13.15535 |
| SFRP1 | -5.39382 | 5.86416 | -10.9954 | 5.50E-10 | 1.62E-07 | 13.11236 |
| SFRP5 | -5.78338 | 6.701978 | -10.9324 | 6.08E-10 | 1.74E-07 | 13.01444 |
| SCN3B | -3.01691 | 7.151348 | -10.8753 | 6.66E-10 | 1.86E-07 | 12.92512 |
| PPP1R1A | -6.29696 | 6.204226 | -10.7268 | 8.46E-10 | 2.21E-07 | 12.69129 |
| HIF3A | -4.58585 | 9.651393 | -10.7068 | 8.73E-10 | 2.23E-07 | 12.65967 |
| NTRK3 | -2.094 | 6.109659 | -10.6935 | 8.92E-10 | 2.23E-07 | 12.63852 |
| SYT4 | -3.06021 | 6.045464 | -10.6564 | 9.48E-10 | 2.32E-07 | 12.57947 |
| SLIT3 | -3.43988 | 8.464678 | -10.6228 | 1.00E-09 | 2.35E-07 | 12.52597 |
| ULBP3 | 2.000553 | 3.926624 | 10.561 | 1.11E-09 | 2.55E-07 | 12.42712 |
| DNER | -4.00036 | 4.339727 | -10.5189 | 1.19E-09 | 2.63E-07 | 12.35948 |
| C7 | -5.65426 | 8.333042 | -10.4952 | 1.23E-09 | 2.63E-07 | 12.32132 |
| MAP2 | -2.43439 | 3.435544 | -10.4216 | 1.39E-09 | 2.82E-07 | 12.2025 |
| SLC25A34 | -2.34049 | 9.202539 | -10.4161 | 1.40E-09 | 2.82E-07 | 12.19347 |
| NAP1L2 | -4.15346 | 6.741353 | -10.4114 | 1.42E-09 | 2.82E-07 | 12.18591 |
| GNG7 | -2.12815 | 8.909047 | -10.3059 | 1.69E-09 | 3.25E-07 | 12.01414 |
| PLEKHS1 | 4.349447 | 8.877709 | 10.17751 | 2.09E-09 | 3.89E-07 | 11.80336 |
| RYR3 | -4.91772 | 7.480785 | -10.1566 | 2.16E-09 | 3.91E-07 | 11.76877 |
| CMA1 | -3.91657 | 4.837282 | -10.1483 | 2.19E-09 | 3.91E-07 | 11.75515 |
| CLEC3B | -3.85054 | 8.601553 | -10.0821 | 2.45E-09 | 4.19E-07 | 11.64533 |
| COL11A1 | 4.60254 | 4.90146 | 10.06538 | 2.52E-09 | 4.19E-07 | 11.61755 |
| PUS7 | 2.017616 | 10.41574 | 10.0578 | 2.56E-09 | 4.19E-07 | 11.60492 |
| TMEM100 | -3.6631 | 8.118286 | -10.0475 | 2.60E-09 | 4.19E-07 | 11.58775 |
| IL11RA | -2.17192 | 10.87426 | -9.96736 | 2.98E-09 | 4.62E-07 | 11.45383 |
| CLU | -3.00136 | 6.800728 | -9.96553 | 2.99E-09 | 4.62E-07 | 11.45076 |
| RCAN2 | -2.37509 | 10.6169 | -9.94272 | 3.11E-09 | 4.74E-07 | 11.4125 |
| GPER1 | -3.37117 | 9.436635 | -9.90212 | 3.33E-09 | 4.89E-07 | 11.34419 |
| CLDN1 | 5.650322 | 9.12614 | 9.868216 | 3.53E-09 | 5.12E-07 | 11.28701 |
| SIM2 | 4.034321 | 4.407592 | 9.831455 | 3.76E-09 | 5.39E-07 | 11.22484 |
| KIF5C | -3.37928 | 8.832562 | -9.72747 | 4.50E-09 | 6.29E-07 | 11.04804 |
| SNCG | -2.4676 | 6.647095 | -9.69918 | 4.72E-09 | 6.50E-07 | 10.99971 |
| CPNE7 | 4.420711 | 10.13441 | 9.695038 | 4.76E-09 | 6.50E-07 | 10.99262 |
| C19orf48 | 2.274971 | 8.249171 | 9.688108 | 4.81E-09 | 6.50E-07 | 10.98075 |
| SMKR1 | 3.00238 | 6.141927 | 9.636148 | 5.27E-09 | 7.04E-07 | 10.89163 |
| AGTR1 | -5.40121 | 8.260428 | -9.46189 | 7.15E-09 | 9.13E-07 | 10.59018 |
| ANK2 | -3.97133 | 9.982505 | -9.44706 | 7.34E-09 | 9.23E-07 | 10.56434 |
| BMP3 | -5.09068 | 5.916955 | -9.43747 | 7.46E-09 | 9.23E-07 | 10.54761 |
| SNAP91 | -2.46657 | 6.951219 | -9.42703 | 7.60E-09 | 9.30E-07 | 10.52938 |
| HLF | -4.1185 | 8.188891 | -9.37095 | 8.40E-09 | 1.01E-06 | 10.43128 |
| C9orf24 | -2.59079 | 9.170186 | -9.36573 | 8.47E-09 | 1.01E-06 | 10.42212 |
| FABP4 | -6.2045 | 9.53626 | -9.34197 | 8.84E-09 | 1.03E-06 | 10.38042 |
| RIN1 | 2.074342 | 10.14981 | 9.319413 | 9.20E-09 | 1.06E-06 | 10.34075 |
| PRKAA2 | -4.59737 | 6.932724 | -9.31231 | 9.32E-09 | 1.06E-06 | 10.32823 |
| MAP1B | -3.31033 | 11.67852 | -9.19325 | 1.15E-08 | 1.25E-06 | 10.11764 |
| MAL | -4.19553 | 9.76241 | -9.19019 | 1.16E-08 | 1.25E-06 | 10.1122 |
| DMD | -4.49773 | 7.101597 | -9.17877 | 1.18E-08 | 1.26E-06 | 10.09191 |
| TTYH1 | -4.05105 | 7.580731 | -9.16878 | 1.20E-08 | 1.27E-06 | 10.07413 |
| GPR12 | -2.29122 | 4.218848 | -9.16593 | 1.21E-08 | 1.27E-06 | 10.06906 |
| GSTM3 | -3.49116 | 6.196105 | -9.14726 | 1.25E-08 | 1.29E-06 | 10.03579 |
| HS6ST3 | -2.82781 | 3.529283 | -9.14145 | 1.27E-08 | 1.29E-06 | 10.02544 |
| CPNE4 | -2.46011 | 3.431116 | -9.10575 | 1.35E-08 | 1.36E-06 | 9.96167 |
| DPT | -3.76582 | 12.71531 | -9.10498 | 1.35E-08 | 1.36E-06 | 9.960288 |
| GALNT16 | -2.97623 | 3.560159 | -9.06624 | 1.45E-08 | 1.43E-06 | 9.890913 |
| TMEM132C | -3.72465 | 6.326507 | -9.05001 | 1.49E-08 | 1.45E-06 | 9.861766 |
| CHRDL1 | -4.87832 | 9.951311 | -9.04838 | 1.50E-08 | 1.45E-06 | 9.858853 |
| TGFBI | 2.019565 | 12.76805 | 9.029478 | 1.55E-08 | 1.49E-06 | 9.824873 |
| ITGA2 | 2.766763 | 8.640971 | 9.025631 | 1.56E-08 | 1.49E-06 | 9.817952 |
| SLC35F1 | -3.24747 | 7.755637 | -9.00755 | 1.61E-08 | 1.52E-06 | 9.785399 |
| C17orf96 | 2.940865 | 9.682878 | 8.874521 | 2.06E-08 | 1.87E-06 | 9.544542 |
| SNCA | -4.01099 | 6.97725 | -8.86181 | 2.10E-08 | 1.89E-06 | 9.521401 |
| GSTM5 | -3.25925 | 6.347459 | -8.86087 | 2.11E-08 | 1.89E-06 | 9.519686 |
| PRELP | -4.21374 | 6.585385 | -8.85376 | 2.13E-08 | 1.90E-06 | 9.506732 |
| CYP4B1 | -3.422 | 7.636703 | -8.79198 | 2.39E-08 | 2.03E-06 | 9.393895 |
| PTN | -3.05778 | 8.855072 | -8.75612 | 2.56E-08 | 2.13E-06 | 9.32815 |
| LOC100506388 | -2.81478 | 7.287346 | -8.70434 | 2.81E-08 | 2.28E-06 | 9.232909 |
| CCBE1 | -6.41732 | 6.869959 | -8.69014 | 2.89E-08 | 2.31E-06 | 9.206729 |
| MYOT | -4.86292 | 7.242409 | -8.64215 | 3.16E-08 | 2.49E-06 | 9.118046 |
| NBEA | -3.68603 | 8.683797 | -8.64183 | 3.16E-08 | 2.49E-06 | 9.117453 |
| GRIN2D | 3.858888 | 7.732016 | 8.611369 | 3.34E-08 | 2.59E-06 | 9.061 |
| TRIP13 | 2.117265 | 9.244185 | 8.609791 | 3.35E-08 | 2.59E-06 | 9.058072 |
| GPM6B | -3.87421 | 8.737928 | -8.60597 | 3.38E-08 | 2.59E-06 | 9.050987 |
| HAND2 | -4.97002 | 9.518965 | -8.59742 | 3.43E-08 | 2.62E-06 | 9.035112 |
| SGCA | -3.70704 | 9.860533 | -8.5614 | 3.67E-08 | 2.71E-06 | 8.968112 |
| RBPMS2 | -4.80778 | 9.26417 | -8.55387 | 3.72E-08 | 2.71E-06 | 8.954085 |
| GAP43 | -3.16517 | 7.410485 | -8.55303 | 3.73E-08 | 2.71E-06 | 8.952513 |
| TSPAN7 | -2.06388 | 7.223674 | -8.5509 | 3.75E-08 | 2.71E-06 | 8.94854 |
| C15orf62 | 2.759055 | 6.730306 | 8.548885 | 3.76E-08 | 2.71E-06 | 8.944794 |
| ZNF471 | -2.45707 | 6.473453 | -8.54554 | 3.78E-08 | 2.71E-06 | 8.938564 |
| SPARCL1 | -3.50675 | 12.06286 | -8.53399 | 3.87E-08 | 2.74E-06 | 8.917016 |
| PRDM12 | 2.860449 | 4.107889 | 8.493235 | 4.17E-08 | 2.90E-06 | 8.840844 |
| SVEP1 | -3.29437 | 5.829473 | -8.4893 | 4.21E-08 | 2.90E-06 | 8.833471 |
| RBMS3 | -2.38222 | 8.986665 | -8.48766 | 4.22E-08 | 2.90E-06 | 8.830408 |
| ESM1 | 3.88005 | 4.929296 | 8.48716 | 4.22E-08 | 2.90E-06 | 8.82947 |
| KCNIP3 | -2.76782 | 8.799101 | -8.47395 | 4.33E-08 | 2.94E-06 | 8.804712 |
| SETBP1 | -2.3058 | 9.708388 | -8.4656 | 4.40E-08 | 2.97E-06 | 8.78907 |
| DPY19L2 | -2.24213 | 3.596899 | -8.44224 | 4.60E-08 | 3.07E-06 | 8.745211 |
| ZSCAN18 | -2.48846 | 10.50627 | -8.42877 | 4.71E-08 | 3.09E-06 | 8.719893 |
| MPPED2 | -2.71975 | 3.536609 | -8.42698 | 4.73E-08 | 3.09E-06 | 8.716521 |
| FAM107A | -2.05732 | 11.76979 | -8.40831 | 4.90E-08 | 3.18E-06 | 8.681383 |
| CHD5 | -3.32618 | 5.800494 | -8.3585 | 5.39E-08 | 3.44E-06 | 8.587393 |
| SHISA2 | 4.206495 | 5.974727 | 8.34848 | 5.49E-08 | 3.47E-06 | 8.568447 |
| PDE3A | -3.19759 | 10.58788 | -8.34219 | 5.56E-08 | 3.49E-06 | 8.55654 |
| PCDH9 | -4.12839 | 5.528419 | -8.33491 | 5.63E-08 | 3.50E-06 | 8.542759 |
| CBX7 | -2.24597 | 12.61732 | -8.31876 | 5.81E-08 | 3.51E-06 | 8.512169 |
| NDST4 | -2.665 | 3.33895 | -8.31114 | 5.90E-08 | 3.53E-06 | 8.49771 |
| C1QTNF7 | -3.2428 | 6.956887 | -8.26592 | 6.43E-08 | 3.76E-06 | 8.411784 |
| RNF43 | 2.927885 | 9.256401 | 8.263095 | 6.46E-08 | 3.76E-06 | 8.406408 |
| TTLL7 | -3.04828 | 6.693289 | -8.21977 | 7.02E-08 | 4.04E-06 | 8.32381 |
| PTH1R | -2.7808 | 4.178241 | -8.19995 | 7.29E-08 | 4.16E-06 | 8.285926 |
| LAIR2 | 4.033176 | 6.239687 | 8.160773 | 7.86E-08 | 4.42E-06 | 8.210893 |
| FGFBP2 | -3.86628 | 7.009281 | -8.12851 | 8.37E-08 | 4.66E-06 | 8.148939 |
| JAM2 | -2.61546 | 7.816567 | -8.12137 | 8.49E-08 | 4.70E-06 | 8.135208 |
| TRIM29 | 3.199597 | 6.642055 | 8.114591 | 8.60E-08 | 4.74E-06 | 8.12217 |
| CKS2 | 2.653987 | 12.46771 | 8.086128 | 9.08E-08 | 4.92E-06 | 8.067341 |
| MUSTN1 | -2.50863 | 9.75563 | -8.06976 | 9.38E-08 | 5.03E-06 | 8.035767 |
| TNS1 | -4.18186 | 13.21375 | -8.06746 | 9.42E-08 | 5.03E-06 | 8.031318 |
| SORBS1 | -3.90154 | 11.19007 | -8.05234 | 9.70E-08 | 5.16E-06 | 8.002099 |
| TMTC1 | -2.41606 | 9.717677 | -8.04319 | 9.87E-08 | 5.23E-06 | 7.984421 |
| TMEM108 | -2.4026 | 3.648046 | -8.02697 | 1.02E-07 | 5.35E-06 | 7.953016 |
| BAALC | -2.54114 | 5.715751 | -8.02212 | 1.03E-07 | 5.37E-06 | 7.943622 |
| PDE5A | -2.66112 | 12.36699 | -8.00145 | 1.07E-07 | 5.52E-06 | 7.903553 |
| PTCHD1 | -5.21321 | 4.674498 | -7.95509 | 1.17E-07 | 5.96E-06 | 7.813451 |
| TCEAL2 | -5.95327 | 6.542635 | -7.95012 | 1.18E-07 | 5.97E-06 | 7.803775 |
| PI16 | -4.85425 | 9.124179 | -7.94932 | 1.19E-07 | 5.97E-06 | 7.802218 |
| ITGA7 | -3.39191 | 9.392272 | -7.94143 | 1.20E-07 | 6.02E-06 | 7.786856 |
| NLGN4X | -2.0096 | 7.327636 | -7.92919 | 1.23E-07 | 6.14E-06 | 7.762983 |
| CNTN2 | -2.11867 | 6.233817 | -7.91572 | 1.27E-07 | 6.28E-06 | 7.736707 |
| TF | -3.77558 | 4.583922 | -7.91033 | 1.28E-07 | 6.32E-06 | 7.726182 |
| SYN2 | -2.67961 | 3.606746 | -7.90002 | 1.31E-07 | 6.42E-06 | 7.706035 |
| FAM150A | 3.497683 | 6.846597 | 7.860818 | 1.41E-07 | 6.79E-06 | 7.629314 |
| PTPRZ1 | -3.6114 | 4.322013 | -7.86066 | 1.41E-07 | 6.79E-06 | 7.629005 |
| PPP2R3A | -2.46056 | 9.767389 | -7.85224 | 1.43E-07 | 6.88E-06 | 7.612499 |
| IL32 | 2.062334 | 11.90846 | 7.824288 | 1.52E-07 | 7.15E-06 | 7.557627 |
| MACC1 | 3.245631 | 5.999454 | 7.813894 | 1.55E-07 | 7.27E-06 | 7.537195 |
| KCNMB2 | -2.52754 | 3.610888 | -7.8097 | 1.56E-07 | 7.30E-06 | 7.528952 |
| CHL1 | -2.6669 | 8.22619 | -7.79718 | 1.60E-07 | 7.45E-06 | 7.504302 |
| TMEM151B | -4.40243 | 4.647525 | -7.78933 | 1.62E-07 | 7.54E-06 | 7.488853 |
| AP1S2 | -2.01727 | 8.997269 | -7.78318 | 1.64E-07 | 7.57E-06 | 7.476722 |
| KCNIP1 | -2.61013 | 5.015832 | -7.74067 | 1.79E-07 | 8.14E-06 | 7.39283 |
| ASXL3 | -3.25897 | 6.950544 | -7.73199 | 1.82E-07 | 8.22E-06 | 7.375671 |
| C19orf45 | 3.166799 | 4.719838 | 7.726662 | 1.84E-07 | 8.28E-06 | 7.365123 |
| CPEB1 | -2.06146 | 6.602187 | -7.68681 | 1.99E-07 | 8.82E-06 | 7.286159 |
| PLN | -4.56032 | 8.979779 | -7.67221 | 2.05E-07 | 8.97E-06 | 7.257168 |
| BEND5 | -2.85082 | 7.698622 | -7.67111 | 2.05E-07 | 8.97E-06 | 7.254984 |
| AK5 | -2.72378 | 4.765029 | -7.64511 | 2.16E-07 | 9.31E-06 | 7.20329 |
| GUCA2B | -5.56166 | 9.184695 | -7.64504 | 2.16E-07 | 9.31E-06 | 7.203154 |
| CTSG | -3.96355 | 8.780441 | -7.63766 | 2.19E-07 | 9.38E-06 | 7.188462 |
| APBB1 | -2.26037 | 11.22647 | -7.6375 | 2.20E-07 | 9.38E-06 | 7.188146 |
| ASPA | -3.39866 | 6.726861 | -7.62316 | 2.26E-07 | 9.58E-06 | 7.159575 |
| CDH19 | -2.80548 | 9.079657 | -7.61194 | 2.31E-07 | 9.73E-06 | 7.13721 |
| SLC7A5 | 2.265801 | 10.81203 | 7.603062 | 2.35E-07 | 9.87E-06 | 7.119488 |
| CRLF1 | -2.09447 | 8.263805 | -7.59837 | 2.37E-07 | 9.93E-06 | 7.110121 |
| CNTNAP3 | -2.83702 | 6.269374 | -7.54394 | 2.65E-07 | 1.07E-05 | 7.00124 |
| GALNT6 | 2.274772 | 6.485708 | 7.536284 | 2.69E-07 | 1.07E-05 | 6.985885 |
| PCOLCE2 | -4.19195 | 9.614052 | -7.53231 | 2.71E-07 | 1.08E-05 | 6.977912 |
| FAM189A2 | -3.46866 | 8.800886 | -7.52883 | 2.73E-07 | 1.08E-05 | 6.97094 |
| GPX3 | -3.48339 | 7.750958 | -7.51936 | 2.78E-07 | 1.09E-05 | 6.951932 |
| C16orf45 | -2.55761 | 10.59228 | -7.50269 | 2.88E-07 | 1.12E-05 | 6.918432 |
| DPP6 | -5.49063 | 7.622979 | -7.49679 | 2.91E-07 | 1.13E-05 | 6.906568 |
| PRRT2 | -2.61643 | 6.751993 | -7.49366 | 2.93E-07 | 1.13E-05 | 6.900276 |
| SLC2A4 | -3.8829 | 8.119784 | -7.48777 | 2.96E-07 | 1.14E-05 | 6.888428 |
| ABCG2 | -3.61285 | 4.830941 | -7.47936 | 3.02E-07 | 1.15E-05 | 6.871508 |
| TCEAL5 | -2.07342 | 8.447652 | -7.47008 | 3.07E-07 | 1.16E-05 | 6.852808 |
| TNS2 | -2.16597 | 12.61138 | -7.45691 | 3.16E-07 | 1.18E-05 | 6.826279 |
| CCDC85A | -2.56431 | 6.950341 | -7.45581 | 3.16E-07 | 1.18E-05 | 6.824062 |
| MICU3 | -2.32969 | 7.692211 | -7.4518 | 3.19E-07 | 1.19E-05 | 6.815965 |
| P3H2 | -2.99321 | 8.479195 | -7.45137 | 3.19E-07 | 1.19E-05 | 6.815095 |
| FAM84B | 2.080422 | 7.678101 | 7.432248 | 3.32E-07 | 1.22E-05 | 6.7765 |
| PRR11 | 2.538403 | 9.476727 | 7.428795 | 3.34E-07 | 1.22E-05 | 6.769524 |
| LMO3 | -4.83187 | 8.148991 | -7.42871 | 3.34E-07 | 1.22E-05 | 6.769358 |
| PRICKLE2 | -2.7491 | 8.932711 | -7.41966 | 3.40E-07 | 1.23E-05 | 6.751068 |
| DGKB | -3.1843 | 4.277384 | -7.41883 | 3.41E-07 | 1.23E-05 | 6.749384 |
| KCNE2 | -2.41476 | 3.62806 | -7.40649 | 3.49E-07 | 1.25E-05 | 6.724425 |
| CCDC69 | -3.14571 | 11.64092 | -7.39782 | 3.56E-07 | 1.25E-05 | 6.706868 |
| CSRNP3 | -2.45063 | 6.456691 | -7.39107 | 3.61E-07 | 1.26E-05 | 6.693207 |
| ZNF483 | -2.95601 | 4.244976 | -7.37191 | 3.75E-07 | 1.30E-05 | 6.654363 |
| LOC101928841 | -4.53833 | 5.812217 | -7.36444 | 3.81E-07 | 1.32E-05 | 6.639218 |
| USP2 | -2.51816 | 6.593426 | -7.36425 | 3.81E-07 | 1.32E-05 | 6.638823 |
| DCHS2 | -2.90878 | 4.193795 | -7.35966 | 3.84E-07 | 1.32E-05 | 6.62952 |
| PDE7B | -2.14315 | 6.209023 | -7.32648 | 4.11E-07 | 1.39E-05 | 6.562071 |
| PREX2 | -2.14678 | 6.974265 | -7.26431 | 4.67E-07 | 1.53E-05 | 6.43531 |
| FBXO41 | 2.074657 | 8.805498 | 7.264053 | 4.67E-07 | 1.53E-05 | 6.434793 |
| NGB | -4.6444 | 6.62437 | -7.26339 | 4.68E-07 | 1.53E-05 | 6.433429 |
| MMP11 | 2.487074 | 6.201376 | 7.248992 | 4.82E-07 | 1.56E-05 | 6.404003 |
| LRFN5 | -3.19557 | 4.617859 | -7.23702 | 4.94E-07 | 1.59E-05 | 6.37951 |
| FILIP1 | -3.55429 | 7.192392 | -7.22838 | 5.03E-07 | 1.61E-05 | 6.361813 |
| KCNN3 | -3.39953 | 7.155942 | -7.21905 | 5.12E-07 | 1.63E-05 | 6.342701 |
| FXYD6 | -3.58498 | 13.32497 | -7.21282 | 5.19E-07 | 1.64E-05 | 6.329919 |
| LIMS2 | -3.72057 | 13.90057 | -7.2126 | 5.19E-07 | 1.64E-05 | 6.329482 |
| CRYAB | -4.42538 | 12.7855 | -7.20134 | 5.31E-07 | 1.67E-05 | 6.306387 |
| SLIT2 | -3.11889 | 8.486666 | -7.16789 | 5.69E-07 | 1.77E-05 | 6.237657 |
| FGL2 | -2.25167 | 11.3101 | -7.1635 | 5.74E-07 | 1.78E-05 | 6.228634 |
| MYH11 | -4.90109 | 11.45286 | -7.15074 | 5.90E-07 | 1.81E-05 | 6.202373 |
| CHODL | -2.08751 | 8.695791 | -7.14741 | 5.94E-07 | 1.81E-05 | 6.195507 |
| FLNC | -4.6053 | 11.98365 | -7.14666 | 5.95E-07 | 1.81E-05 | 6.193971 |
| RERGL | -4.48404 | 7.047874 | -7.13488 | 6.09E-07 | 1.84E-05 | 6.169697 |
| FAM178B | -2.36091 | 4.898726 | -7.12454 | 6.22E-07 | 1.87E-05 | 6.148379 |
| PHOX2B | -2.89652 | 5.784931 | -7.11124 | 6.40E-07 | 1.91E-05 | 6.120942 |
| FAM46B | -4.97279 | 10.34094 | -7.09486 | 6.62E-07 | 1.96E-05 | 6.087094 |
| DMRTC1 | -3.13782 | 3.963355 | -7.09404 | 6.63E-07 | 1.96E-05 | 6.085401 |
| COL21A1 | -3.31773 | 5.311302 | -7.07081 | 6.95E-07 | 2.05E-05 | 6.03736 |
| MCIDAS | 3.137356 | 3.496461 | 7.06818 | 6.99E-07 | 2.05E-05 | 6.031911 |
| SORD | 2.267856 | 4.175125 | 7.060302 | 7.11E-07 | 2.07E-05 | 6.015595 |
| C1orf95 | -2.3914 | 7.198641 | -7.05606 | 7.17E-07 | 2.09E-05 | 6.006799 |
| PBX1 | -2.39974 | 12.42125 | -7.05352 | 7.21E-07 | 2.09E-05 | 6.001545 |
| GDF15 | 2.687997 | 8.921341 | 7.051368 | 7.24E-07 | 2.09E-05 | 5.997085 |
| TLX1 | 4.332149 | 5.399591 | 7.045138 | 7.34E-07 | 2.09E-05 | 5.984168 |
| GPR17 | -2.95929 | 4.469131 | -7.04449 | 7.35E-07 | 2.09E-05 | 5.982817 |
| BHMT2 | -3.61694 | 4.865362 | -7.02579 | 7.64E-07 | 2.15E-05 | 5.944018 |
| SOX15 | -3.15843 | 7.307071 | -7.01504 | 7.81E-07 | 2.19E-05 | 5.921695 |
| FIGF | -3.88641 | 5.988474 | -7.01451 | 7.82E-07 | 2.19E-05 | 5.9206 |
| FAM13C | -2.92716 | 8.184042 | -7.01263 | 7.85E-07 | 2.19E-05 | 5.916685 |
| SMIM5 | -2.07179 | 8.728127 | -7.00362 | 8.00E-07 | 2.21E-05 | 5.897958 |
| CTNND2 | -3.18327 | 3.844257 | -6.99989 | 8.06E-07 | 2.22E-05 | 5.89021 |
| AR | -3.02502 | 6.814875 | -6.98422 | 8.33E-07 | 2.26E-05 | 5.857606 |
| RAP1A | -2.4172 | 9.829764 | -6.97779 | 8.44E-07 | 2.28E-05 | 5.844214 |
| GNAO1 | -2.75638 | 6.956777 | -6.96365 | 8.69E-07 | 2.33E-05 | 5.814758 |
| PEG3 | -3.36944 | 6.244382 | -6.96293 | 8.70E-07 | 2.33E-05 | 5.813257 |
| FGF13 | -2.70341 | 7.211903 | -6.96053 | 8.75E-07 | 2.33E-05 | 5.808235 |
| ASCL5 | 3.268174 | 5.51251 | 6.955157 | 8.85E-07 | 2.35E-05 | 5.797039 |
| C20orf194 | -2.41672 | 9.555794 | -6.9455 | 9.03E-07 | 2.38E-05 | 5.776886 |
| FXYD6-FXYD2 | -3.09827 | 7.547498 | -6.93562 | 9.22E-07 | 2.42E-05 | 5.756268 |
| FGF7 | -2.84483 | 7.327171 | -6.9304 | 9.32E-07 | 2.44E-05 | 5.745362 |
| LRCH2 | -2.23645 | 7.971772 | -6.92901 | 9.34E-07 | 2.44E-05 | 5.742464 |
| TRIM63 | -3.18204 | 5.229092 | -6.9266 | 9.39E-07 | 2.45E-05 | 5.737412 |
| PIRT | -3.39485 | 5.919149 | -6.9177 | 9.57E-07 | 2.47E-05 | 5.718814 |
| ANGPTL7 | -4.83772 | 6.370085 | -6.91714 | 9.58E-07 | 2.47E-05 | 5.717644 |
| BRCA2 | 2.067796 | 7.36687 | 6.908282 | 9.76E-07 | 2.47E-05 | 5.699119 |
| ANLN | 3.10224 | 6.233394 | 6.908035 | 9.76E-07 | 2.47E-05 | 5.698601 |
| SLC26A7 | -2.62827 | 4.944417 | -6.90375 | 9.85E-07 | 2.48E-05 | 5.689644 |
| PARD6B | 2.142449 | 8.973663 | 6.902906 | 9.87E-07 | 2.48E-05 | 5.687868 |
| MYL2 | -2.04 | 4.276219 | -6.88355 | 1.03E-06 | 2.57E-05 | 5.647329 |
| KISS1 | 2.37318 | 6.823703 | 6.878285 | 1.04E-06 | 2.59E-05 | 5.636299 |
| CLIP4 | -2.52275 | 7.74968 | -6.87609 | 1.04E-06 | 2.60E-05 | 5.631695 |
| ATOH8 | -2.25153 | 10.85866 | -6.87403 | 1.05E-06 | 2.60E-05 | 5.62738 |
| AKR1C1 | -3.21271 | 9.764144 | -6.8617 | 1.08E-06 | 2.66E-05 | 5.601505 |
| KANK2 | -2.74927 | 13.58166 | -6.85811 | 1.08E-06 | 2.66E-05 | 5.593974 |
| JAZF1 | -2.25782 | 9.562666 | -6.85043 | 1.10E-06 | 2.70E-05 | 5.577848 |
| CES1 | -3.34197 | 12.40036 | -6.84798 | 1.11E-06 | 2.71E-05 | 5.572709 |
| MSRB3 | -3.27953 | 10.93736 | -6.83407 | 1.14E-06 | 2.76E-05 | 5.543474 |
| ECT2 | 2.583248 | 9.382931 | 6.822616 | 1.17E-06 | 2.81E-05 | 5.519395 |
| RADIL | -3.01861 | 7.618617 | -6.82173 | 1.17E-06 | 2.81E-05 | 5.517521 |
| MAD2L1 | 2.668854 | 9.360922 | 6.821571 | 1.17E-06 | 2.81E-05 | 5.517197 |
| ZBTB16 | -3.74089 | 12.37963 | -6.82135 | 1.17E-06 | 2.81E-05 | 5.516732 |
| SGCE | -2.52321 | 11.00764 | -6.81395 | 1.19E-06 | 2.84E-05 | 5.501168 |
| DES | -4.92672 | 12.90973 | -6.79722 | 1.23E-06 | 2.93E-05 | 5.465915 |
| SHCBP1 | 2.403732 | 8.68452 | 6.795794 | 1.24E-06 | 2.93E-05 | 5.462921 |
| THSD7A | -2.20266 | 6.744797 | -6.78978 | 1.25E-06 | 2.95E-05 | 5.450251 |
| ST6GAL2 | -3.62233 | 6.828354 | -6.78726 | 1.26E-06 | 2.95E-05 | 5.444933 |
| SLC26A10 | -2.92371 | 9.925809 | -6.78464 | 1.27E-06 | 2.96E-05 | 5.439397 |
| CTSV | 2.416446 | 4.12581 | 6.782926 | 1.27E-06 | 2.97E-05 | 5.435791 |
| ABCA9 | -2.60523 | 6.641947 | -6.76086 | 1.33E-06 | 3.10E-05 | 5.389212 |
| UNC5CL | 2.36085 | 8.832876 | 6.743284 | 1.38E-06 | 3.18E-05 | 5.35208 |
| JPH2 | -4.3157 | 7.200336 | -6.74117 | 1.39E-06 | 3.19E-05 | 5.347617 |
| DSEL | -2.21756 | 6.691856 | -6.739 | 1.39E-06 | 3.19E-05 | 5.343018 |
| GNAZ | -2.17788 | 7.37747 | -6.73503 | 1.41E-06 | 3.21E-05 | 5.334615 |
| RASL12 | -2.21457 | 10.94174 | -6.7327 | 1.41E-06 | 3.21E-05 | 5.3297 |
| ADIPOQ | -4.92787 | 5.699247 | -6.72924 | 1.42E-06 | 3.23E-05 | 5.32238 |
| OR51E2 | -2.81885 | 6.804991 | -6.7274 | 1.43E-06 | 3.23E-05 | 5.318473 |
| FANCB | 2.960452 | 4.200643 | 6.726519 | 1.43E-06 | 3.23E-05 | 5.316613 |
| SCRG1 | -5.45338 | 6.883167 | -6.72052 | 1.45E-06 | 3.26E-05 | 5.303923 |
| ACKR1 | -2.57582 | 11.77861 | -6.7033 | 1.50E-06 | 3.35E-05 | 5.267436 |
| DPYSL5 | -3.33535 | 3.746522 | -6.69975 | 1.51E-06 | 3.35E-05 | 5.259909 |
| RNF180 | -3.04871 | 4.892372 | -6.69175 | 1.54E-06 | 3.40E-05 | 5.242938 |
| SVIL | -2.61037 | 11.95552 | -6.68242 | 1.57E-06 | 3.45E-05 | 5.223143 |
| TMOD2 | -2.26444 | 9.361061 | -6.67683 | 1.59E-06 | 3.48E-05 | 5.211268 |
| SALL2 | -2.6319 | 6.123043 | -6.67161 | 1.61E-06 | 3.50E-05 | 5.200186 |
| RGN | -2.76442 | 8.236941 | -6.6686 | 1.62E-06 | 3.51E-05 | 5.193799 |
| RBM24 | -3.68601 | 5.092124 | -6.66693 | 1.62E-06 | 3.51E-05 | 5.190242 |
| RGMA | -2.76075 | 10.32785 | -6.65106 | 1.68E-06 | 3.61E-05 | 5.156524 |
| KIAA0408 | -3.48297 | 7.627763 | -6.64449 | 1.70E-06 | 3.65E-05 | 5.142552 |
| ATP2B4 | -3.01723 | 13.20204 | -6.63663 | 1.73E-06 | 3.69E-05 | 5.12582 |
| MGP | -3.37499 | 14.19578 | -6.63408 | 1.74E-06 | 3.70E-05 | 5.120381 |
| ITPKB | -2.00878 | 13.14962 | -6.62213 | 1.79E-06 | 3.78E-05 | 5.094936 |
| MYOCD | -3.7993 | 9.386059 | -6.61647 | 1.81E-06 | 3.82E-05 | 5.082878 |
| KCNMB1 | -4.00648 | 11.62802 | -6.61259 | 1.82E-06 | 3.84E-05 | 5.074605 |
| TFR2 | 2.649349 | 6.787562 | 6.572988 | 1.98E-06 | 4.10E-05 | 4.990093 |
| CAP2 | -2.89825 | 9.782835 | -6.56462 | 2.02E-06 | 4.16E-05 | 4.972213 |
| FGFR1 | -2.451 | 7.531804 | -6.55021 | 2.08E-06 | 4.27E-05 | 4.941377 |
| PARPBP | 2.374767 | 9.300482 | 6.550078 | 2.08E-06 | 4.27E-05 | 4.941104 |
| TMEM132B | -2.07944 | 3.882296 | -6.52796 | 2.19E-06 | 4.44E-05 | 4.89374 |
| CKAP2L | 2.853922 | 4.830903 | 6.526586 | 2.19E-06 | 4.44E-05 | 4.890799 |
| NGFR | -2.38418 | 8.97769 | -6.51076 | 2.27E-06 | 4.58E-05 | 4.856869 |
| HELLS | 2.134707 | 9.506594 | 6.505999 | 2.29E-06 | 4.60E-05 | 4.846658 |
| TCEAL7 | -2.3315 | 6.802954 | -6.47711 | 2.44E-06 | 4.83E-05 | 4.784618 |
| IGFBP6 | -2.94589 | 13.70675 | -6.4586 | 2.54E-06 | 4.99E-05 | 4.744811 |
| AUNIP | 3.582185 | 6.73968 | 6.445914 | 2.61E-06 | 5.07E-05 | 4.71751 |
| ITIH5 | -2.79608 | 7.83862 | -6.43283 | 2.68E-06 | 5.17E-05 | 4.689325 |
| MAP6 | -2.32057 | 8.245007 | -6.42808 | 2.71E-06 | 5.21E-05 | 4.679081 |
| BOC | -2.65457 | 8.28153 | -6.41681 | 2.78E-06 | 5.29E-05 | 4.654798 |
| CLDN14 | 2.336411 | 6.435111 | 6.416482 | 2.78E-06 | 5.29E-05 | 4.654083 |
| DBF4 | 2.190035 | 4.39212 | 6.414938 | 2.79E-06 | 5.29E-05 | 4.650752 |
| PTGIS | -4.11439 | 10.8235 | -6.41249 | 2.80E-06 | 5.29E-05 | 4.645463 |
| ONECUT2 | 2.334837 | 3.421069 | 6.412003 | 2.80E-06 | 5.29E-05 | 4.64442 |
| TAGLN | -3.26205 | 14.19096 | -6.41117 | 2.81E-06 | 5.29E-05 | 4.642631 |
| ARHGAP10 | -2.29213 | 11.59215 | -6.40575 | 2.84E-06 | 5.32E-05 | 4.630936 |
| CCNO | 4.410234 | 8.886717 | 6.395643 | 2.91E-06 | 5.40E-05 | 4.609105 |
| RHAG | -2.12318 | 2.991905 | -6.39041 | 2.94E-06 | 5.46E-05 | 4.597796 |
| DYNC1I1 | -3.91995 | 7.810581 | -6.38298 | 2.99E-06 | 5.53E-05 | 4.581757 |
| AXIN2 | 2.198303 | 11.20468 | 6.37078 | 3.07E-06 | 5.63E-05 | 4.555371 |
| VN1R1 | -2.35544 | 4.331378 | -6.3504 | 3.20E-06 | 5.86E-05 | 4.511275 |
| CPXM2 | -3.4971 | 8.357953 | -6.34716 | 3.23E-06 | 5.88E-05 | 4.504259 |
| RNF150 | -4.0487 | 6.51569 | -6.34232 | 3.26E-06 | 5.90E-05 | 4.49377 |
| CDKL2 | -2.32549 | 3.629094 | -6.33728 | 3.30E-06 | 5.96E-05 | 4.482844 |
| GNG3 | -3.28023 | 7.12613 | -6.32612 | 3.38E-06 | 6.09E-05 | 4.458654 |
| LIPG | 3.077392 | 9.063199 | 6.32475 | 3.39E-06 | 6.10E-05 | 4.455685 |
| CBX6 | -2.08669 | 11.84006 | -6.31266 | 3.48E-06 | 6.23E-05 | 4.429454 |
| LHFPL4 | -2.03943 | 6.202304 | -6.3097 | 3.50E-06 | 6.24E-05 | 4.423039 |
| MEIS1 | -2.8652 | 8.464484 | -6.2903 | 3.65E-06 | 6.39E-05 | 4.380896 |
| CTSF | -2.11708 | 12.56275 | -6.26504 | 3.86E-06 | 6.67E-05 | 4.325982 |
| PTTG1 | 2.104414 | 9.297292 | 6.254174 | 3.95E-06 | 6.79E-05 | 4.302325 |
| DIP2C | -2.02636 | 7.871907 | -6.24804 | 4.00E-06 | 6.86E-05 | 4.288976 |
| PI15 | -3.15459 | 7.799039 | -6.24387 | 4.04E-06 | 6.90E-05 | 4.27989 |
| MYLK | -3.18054 | 8.920256 | -6.24205 | 4.06E-06 | 6.92E-05 | 4.275928 |
| C1QTNF9 | -3.19224 | 3.710742 | -6.22927 | 4.17E-06 | 7.08E-05 | 4.24806 |
| MST1L | -3.73685 | 4.466663 | -6.2134 | 4.32E-06 | 7.25E-05 | 4.213447 |
| SLCO4A1 | 2.512385 | 11.36197 | 6.200991 | 4.44E-06 | 7.38E-05 | 4.186358 |
| REEP2 | -3.21872 | 8.786278 | -6.19793 | 4.47E-06 | 7.42E-05 | 4.179664 |
| LGR5 | 4.446267 | 7.693399 | 6.194726 | 4.50E-06 | 7.43E-05 | 4.172672 |
| FOLR2 | -2.03023 | 11.21078 | -6.17159 | 4.73E-06 | 7.74E-05 | 4.122107 |
| TRIM59 | 2.148601 | 5.411805 | 6.168662 | 4.76E-06 | 7.78E-05 | 4.115693 |
| KRT6B | 3.589806 | 8.427258 | 6.165669 | 4.79E-06 | 7.81E-05 | 4.109144 |
| PTGER3 | -2.47048 | 6.84855 | -6.16008 | 4.85E-06 | 7.86E-05 | 4.096917 |
| ADH1C | -3.1694 | 10.59603 | -6.15711 | 4.88E-06 | 7.91E-05 | 4.090421 |
| DACT3 | -3.47176 | 10.98343 | -6.15115 | 4.95E-06 | 7.98E-05 | 4.077367 |
| NFASC | -2.03609 | 5.754856 | -6.1511 | 4.95E-06 | 7.98E-05 | 4.077253 |
| HSPB2 | -2.51108 | 10.0666 | -6.14857 | 4.98E-06 | 8.01E-05 | 4.071714 |
| APOBEC1 | 4.555178 | 7.734224 | 6.144657 | 5.02E-06 | 8.05E-05 | 4.063141 |
| FERMT2 | -2.89678 | 11.08568 | -6.14198 | 5.05E-06 | 8.08E-05 | 4.057269 |
| TMEM179 | -3.5523 | 6.73452 | -6.13866 | 5.09E-06 | 8.10E-05 | 4.05001 |
| TTC28 | -2.29758 | 4.382035 | -6.13149 | 5.17E-06 | 8.20E-05 | 4.034292 |
| PITX1 | 2.545212 | 11.60457 | 6.115685 | 5.35E-06 | 8.38E-05 | 3.999625 |
| MEIS2 | -3.18921 | 7.717298 | -6.1126 | 5.39E-06 | 8.43E-05 | 3.992864 |
| KLB | -3.53834 | 5.239226 | -6.10682 | 5.45E-06 | 8.50E-05 | 3.980178 |
| NLGN3 | -2.14362 | 6.054013 | -6.10382 | 5.49E-06 | 8.54E-05 | 3.973579 |
| NUPR1L | -2.38538 | 6.269327 | -6.09616 | 5.58E-06 | 8.64E-05 | 3.956752 |
| SUSD5 | -2.56643 | 5.803259 | -6.07753 | 5.82E-06 | 8.90E-05 | 3.915816 |
| SSTR2 | -2.02961 | 3.921944 | -6.06576 | 5.97E-06 | 9.10E-05 | 3.889929 |
| ALDH1A1 | -2.71781 | 10.99433 | -6.05817 | 6.07E-06 | 9.22E-05 | 3.873232 |
| GRIK1 | -2.77723 | 3.934114 | -6.05727 | 6.08E-06 | 9.22E-05 | 3.871257 |
| NFATC4 | -2.63574 | 8.414076 | -6.05318 | 6.14E-06 | 9.26E-05 | 3.862254 |
| TUBB2B | -3.08119 | 7.952437 | -6.04945 | 6.19E-06 | 9.31E-05 | 3.854038 |
| STEAP4 | -2.69625 | 9.24834 | -6.04544 | 6.24E-06 | 9.36E-05 | 3.845206 |
| CXCL1 | 2.851486 | 4.852661 | 6.044116 | 6.26E-06 | 9.37E-05 | 3.842292 |
| LRAT | -3.4268 | 3.844622 | -6.03518 | 6.39E-06 | 9.52E-05 | 3.822609 |
| C2orf74 | -2.19175 | 9.347706 | -6.02746 | 6.50E-06 | 9.64E-05 | 3.80559 |
| APELA | 3.054401 | 3.731207 | 6.022591 | 6.57E-06 | 9.71E-05 | 3.794856 |
| IQGAP3 | 2.164168 | 10.56602 | 6.01429 | 6.69E-06 | 9.79E-05 | 3.776547 |
| ZFPM2 | -2.66256 | 7.613323 | -6.01144 | 6.73E-06 | 9.84E-05 | 3.770267 |
| PLK1 | 2.382748 | 11.54706 | 5.991931 | 7.03E-06 | 0.000102 | 3.727195 |
| RECQL4 | 2.141415 | 10.6637 | 5.987976 | 7.09E-06 | 0.000103 | 3.718459 |
| COL10A1 | 4.686457 | 4.431069 | 5.983411 | 7.16E-06 | 0.000103 | 3.708374 |
| DPEP1 | 3.34695 | 7.92758 | 5.982607 | 7.17E-06 | 0.000103 | 3.706597 |
| ANKRD22 | 2.412028 | 11.12441 | 5.969502 | 7.38E-06 | 0.000105 | 3.677629 |
| SORBS2 | -2.11851 | 9.350614 | -5.95314 | 7.66E-06 | 0.000109 | 3.641435 |
| KCNN4 | 2.512742 | 12.90076 | 5.926252 | 8.13E-06 | 0.000115 | 3.581893 |
| PGPEP1L | -2.87217 | 3.681939 | -5.92577 | 8.14E-06 | 0.000115 | 3.580823 |
| NPR1 | -2.58032 | 9.951966 | -5.91179 | 8.39E-06 | 0.000118 | 3.549831 |
| CENPE | 2.135142 | 7.99792 | 5.909684 | 8.43E-06 | 0.000118 | 3.545161 |
| TPM1 | -3.11529 | 14.14222 | -5.90702 | 8.48E-06 | 0.000118 | 3.539249 |
| TACSTD2 | 3.061192 | 10.33779 | 5.898127 | 8.65E-06 | 0.00012 | 3.519521 |
| TAC1 | -2.45076 | 6.959971 | -5.88068 | 8.99E-06 | 0.000123 | 3.480789 |
| CDC25C | 2.170909 | 3.880277 | 5.875493 | 9.10E-06 | 0.000125 | 3.469263 |
| CTF1 | -2.47882 | 8.41033 | -5.87086 | 9.19E-06 | 0.000126 | 3.458961 |
| ARHGEF4 | -3.12785 | 9.789097 | -5.84752 | 9.68E-06 | 0.00013 | 3.407081 |
| SCN9A | -2.10015 | 7.034746 | -5.83561 | 9.94E-06 | 0.000133 | 3.380569 |
| TLCD1 | 2.203913 | 10.11928 | 5.817947 | 1.03E-05 | 0.000137 | 3.341232 |
| CCDC80 | -2.20005 | 8.966784 | -5.81312 | 1.05E-05 | 0.000138 | 3.330466 |
| CCL20 | 3.83258 | 9.044481 | 5.783662 | 1.12E-05 | 0.000146 | 3.264782 |
| ADH1A | -3.18541 | 12.91126 | -5.78366 | 1.12E-05 | 0.000146 | 3.264771 |
| ETV7 | 2.184737 | 10.06614 | 5.781117 | 1.12E-05 | 0.000146 | 3.259102 |
| F12 | 2.629951 | 10.46277 | 5.772632 | 1.14E-05 | 0.000149 | 3.24016 |
| CRMP1 | -2.4696 | 8.519811 | -5.74117 | 1.23E-05 | 0.000157 | 3.169867 |
| TRIM9 | -2.2844 | 7.680297 | -5.73908 | 1.23E-05 | 0.000157 | 3.165187 |
| SLITRK3 | -2.71841 | 5.72473 | -5.73817 | 1.24E-05 | 0.000157 | 3.163147 |
| HPSE2 | -2.28595 | 7.639826 | -5.7341 | 1.25E-05 | 0.000158 | 3.154036 |
| SLCO1B3 | 5.401148 | 5.898175 | 5.730114 | 1.26E-05 | 0.000159 | 3.145128 |
| CELSR3 | 2.085841 | 9.144748 | 5.722412 | 1.28E-05 | 0.000162 | 3.127893 |
| CYP2S1 | 2.442741 | 12.76939 | 5.708776 | 1.32E-05 | 0.000166 | 3.097364 |
| CAV2 | -2.14506 | 10.53651 | -5.70815 | 1.32E-05 | 0.000166 | 3.095951 |
| EPSTI1 | 2.14525 | 8.193322 | 5.705311 | 1.33E-05 | 0.000167 | 3.089602 |
| SH3BGR | -2.44418 | 8.877148 | -5.70274 | 1.34E-05 | 0.000168 | 3.083843 |
| CRTAP | -2.05638 | 8.930262 | -5.70087 | 1.34E-05 | 0.000168 | 3.079661 |
| CAMK2B | -2.05297 | 4.358286 | -5.69369 | 1.37E-05 | 0.00017 | 3.063574 |
| EFS | -2.00278 | 9.506889 | -5.68489 | 1.39E-05 | 0.000173 | 3.043843 |
| COLGALT2 | -3.38256 | 9.041565 | -5.68264 | 1.40E-05 | 0.000173 | 3.038807 |
| MMP1 | 3.143517 | 6.26316 | 5.671877 | 1.44E-05 | 0.000177 | 3.014656 |
| SLC7A14 | -2.89104 | 3.746771 | -5.66945 | 1.44E-05 | 0.000178 | 3.009222 |
| NEK2 | 2.468506 | 8.65828 | 5.667395 | 1.45E-05 | 0.000178 | 3.004602 |
| RBPMS | -2.08002 | 11.93315 | -5.66478 | 1.46E-05 | 0.000179 | 2.998738 |
| BUB1 | 2.455062 | 6.266051 | 5.645454 | 1.52E-05 | 0.000186 | 2.955345 |
| KIF18A | 2.833508 | 6.683552 | 5.628478 | 1.58E-05 | 0.000191 | 2.917203 |
| IL21R | 2.057772 | 3.713207 | 5.625046 | 1.59E-05 | 0.000192 | 2.909489 |
| CXCL9 | 3.268833 | 9.399226 | 5.624471 | 1.60E-05 | 0.000192 | 2.908195 |
| CENPI | 2.344712 | 4.695295 | 5.620577 | 1.61E-05 | 0.000193 | 2.89944 |
| CCL24 | 2.228244 | 5.686441 | 5.620013 | 1.61E-05 | 0.000193 | 2.898173 |
| ICOS | 2.960599 | 6.661821 | 5.617241 | 1.62E-05 | 0.000193 | 2.891939 |
| PTRF | -2.38504 | 14.47382 | -5.61612 | 1.63E-05 | 0.000194 | 2.889417 |
| KIF23 | 2.393546 | 8.996303 | 5.613506 | 1.64E-05 | 0.000194 | 2.88354 |
| GRIK2 | -2.7515 | 6.351458 | -5.60961 | 1.65E-05 | 0.000195 | 2.874769 |
| LEPR | -2.02221 | 8.893777 | -5.60569 | 1.67E-05 | 0.000197 | 2.865947 |
| GUCA2A | -3.67827 | 11.76878 | -5.60302 | 1.68E-05 | 0.000197 | 2.859952 |
| NUF2 | 2.06984 | 8.05274 | 5.600251 | 1.69E-05 | 0.000198 | 2.853718 |
| PLA2G5 | -2.01304 | 5.931461 | -5.57657 | 1.78E-05 | 0.000207 | 2.800394 |
| GRIK5 | -2.19661 | 7.487245 | -5.5764 | 1.78E-05 | 0.000207 | 2.800012 |
| RAB6B | -2.4849 | 6.481721 | -5.57375 | 1.79E-05 | 0.000208 | 2.794037 |
| FANCA | 2.192811 | 9.178258 | 5.560546 | 1.84E-05 | 0.000213 | 2.764285 |
| CACNA1C | -2.8381 | 7.997613 | -5.55285 | 1.88E-05 | 0.000216 | 2.746935 |
| TRPC1 | -2.81702 | 9.268238 | -5.55263 | 1.88E-05 | 0.000216 | 2.746426 |
| MT1M | -3.53192 | 8.198136 | -5.54626 | 1.91E-05 | 0.000219 | 2.732067 |
| RGS2 | -3.02013 | 12.40078 | -5.5431 | 1.92E-05 | 0.00022 | 2.724931 |
| NPAS4 | -2.34848 | 7.613011 | -5.54267 | 1.92E-05 | 0.00022 | 2.723969 |
| AJUBA | 2.229343 | 5.86373 | 5.542093 | 1.92E-05 | 0.00022 | 2.722669 |
| AURKA | 2.536121 | 11.33857 | 5.53518 | 1.95E-05 | 0.000223 | 2.707069 |
| CDC20 | 2.374364 | 9.681996 | 5.534214 | 1.96E-05 | 0.000223 | 2.704889 |
| SLC10A4 | -3.34557 | 4.306091 | -5.53411 | 1.96E-05 | 0.000223 | 2.704655 |
| CMYA5 | -2.45118 | 4.910353 | -5.51731 | 2.03E-05 | 0.00023 | 2.666719 |
| REEP1 | -3.04307 | 7.945346 | -5.51701 | 2.04E-05 | 0.00023 | 2.666055 |
| TMEM74 | -2.83616 | 4.928271 | -5.50508 | 2.09E-05 | 0.000235 | 2.639108 |
| WASF3 | -2.25488 | 8.311997 | -5.49643 | 2.13E-05 | 0.000238 | 2.619552 |
| PHYHIP | -2.63303 | 5.470014 | -5.47574 | 2.24E-05 | 0.000247 | 2.572767 |
| SMARCA1 | -2.21916 | 9.03006 | -5.47363 | 2.25E-05 | 0.000248 | 2.567986 |
| MFAP4 | -2.17558 | 14.4127 | -5.46807 | 2.27E-05 | 0.00025 | 2.555416 |
| SOX2 | -3.93159 | 6.128268 | -5.46495 | 2.29E-05 | 0.000251 | 2.548339 |
| RASSF8 | -2.46515 | 9.659148 | -5.46413 | 2.30E-05 | 0.000251 | 2.546482 |
| TESC | 2.941107 | 11.66603 | 5.462265 | 2.30E-05 | 0.000252 | 2.542268 |
| ZG16 | -5.53498 | 7.67153 | -5.46098 | 2.31E-05 | 0.000252 | 2.539351 |
| NPAS3 | -2.2667 | 5.579289 | -5.45602 | 2.34E-05 | 0.000255 | 2.528129 |
| FRRS1L | -2.9766 | 4.289028 | -5.45203 | 2.36E-05 | 0.000257 | 2.519088 |
| SLC17A8 | -2.72789 | 3.590545 | -5.44915 | 2.37E-05 | 0.000258 | 2.512577 |
| CACNA1B | -2.86822 | 3.997446 | -5.44617 | 2.39E-05 | 0.000259 | 2.505821 |
| TPH1 | -3.37149 | 4.943495 | -5.4402 | 2.42E-05 | 0.000262 | 2.492313 |
| PER3 | -2.43804 | 9.069367 | -5.42582 | 2.50E-05 | 0.000267 | 2.459712 |
| E2F7 | 3.197943 | 6.647387 | 5.421972 | 2.53E-05 | 0.000269 | 2.450991 |
| NEIL3 | 2.96344 | 6.198449 | 5.411944 | 2.58E-05 | 0.000273 | 2.428251 |
| SKIDA1 | -2.87359 | 4.315039 | -5.3799 | 2.78E-05 | 0.00029 | 2.355539 |
| ERCC6L | 3.247702 | 7.565125 | 5.377532 | 2.79E-05 | 0.00029 | 2.350153 |
| KCNJ18 | -2.63586 | 8.215829 | -5.35377 | 2.95E-05 | 0.000303 | 2.296171 |
| CSRP1 | -2.82458 | 9.978372 | -5.34649 | 3.00E-05 | 0.000307 | 2.27962 |
| ITGA5 | -3.15241 | 13.48746 | -5.34609 | 3.00E-05 | 0.000307 | 2.278706 |
| AMELX | 3.345839 | 3.7151 | 5.34223 | 3.03E-05 | 0.000309 | 2.269928 |
| KCNA1 | -3.10393 | 4.6261 | -5.33812 | 3.06E-05 | 0.000311 | 2.26057 |
| SLITRK5 | -2.37136 | 5.689516 | -5.32708 | 3.14E-05 | 0.000316 | 2.235464 |
| PCP4L1 | -2.54729 | 4.922134 | -5.32112 | 3.18E-05 | 0.00032 | 2.221911 |
| HSD17B6 | -2.79733 | 10.84825 | -5.3128 | 3.24E-05 | 0.000325 | 2.202962 |
| SCG2 | -2.98103 | 8.369708 | -5.30675 | 3.29E-05 | 0.000328 | 2.189203 |
| SLC6A19 | -3.75242 | 4.841755 | -5.29519 | 3.37E-05 | 0.000334 | 2.162866 |
| C2orf70 | 2.467811 | 7.754229 | 5.293968 | 3.38E-05 | 0.000335 | 2.160084 |
| AKAP6 | -2.52022 | 3.668174 | -5.29044 | 3.41E-05 | 0.000337 | 2.152058 |
| ALPI | -2.43562 | 3.317595 | -5.28503 | 3.45E-05 | 0.00034 | 2.139725 |
| SSC5D | -2.48253 | 10.64994 | -5.27652 | 3.52E-05 | 0.000346 | 2.120318 |
| GINS4 | 2.179448 | 6.593307 | 5.263166 | 3.63E-05 | 0.000353 | 2.089881 |
| SERPINB5 | 3.137089 | 5.051432 | 5.262139 | 3.64E-05 | 0.000353 | 2.087538 |
| HJURP | 2.065562 | 6.802763 | 5.261919 | 3.64E-05 | 0.000353 | 2.087036 |
| DIO2 | 2.415084 | 10.31861 | 5.260797 | 3.65E-05 | 0.000354 | 2.084477 |
| OLFML2A | -2.24273 | 10.99627 | -5.24886 | 3.75E-05 | 0.000362 | 2.057244 |
| EBF1 | -2.41137 | 5.940153 | -5.23336 | 3.89E-05 | 0.000372 | 2.021882 |
| FANCD2OS | 2.045215 | 3.707008 | 5.23103 | 3.91E-05 | 0.000373 | 2.016557 |
| LVRN | -2.38364 | 4.348518 | -5.22887 | 3.93E-05 | 0.000374 | 2.011635 |
| CA7 | -4.90517 | 9.302341 | -5.201 | 4.19E-05 | 0.000394 | 1.947978 |
| CST2 | 3.057842 | 7.247156 | 5.182335 | 4.37E-05 | 0.000408 | 1.905303 |
| KIF24 | 2.115387 | 6.905921 | 5.172442 | 4.47E-05 | 0.000417 | 1.882677 |
| KIF18B | 2.353605 | 10.33648 | 5.171867 | 4.48E-05 | 0.000417 | 1.881363 |
| GALNT15 | -2.22812 | 6.997726 | -5.16281 | 4.57E-05 | 0.000424 | 1.860635 |
| C14orf180 | -3.13809 | 5.183953 | -5.16263 | 4.57E-05 | 0.000424 | 1.860233 |
| HSPA2 | -2.20963 | 11.26782 | -5.1539 | 4.66E-05 | 0.000431 | 1.840247 |
| CD1A | 2.387886 | 5.371284 | 5.130077 | 4.93E-05 | 0.00045 | 1.785714 |
| CENPF | 2.338086 | 8.238166 | 5.12109 | 5.03E-05 | 0.000458 | 1.765129 |
| PRKAR2B | -2.41809 | 7.421779 | -5.11243 | 5.13E-05 | 0.000463 | 1.745293 |
| YPEL4 | -2.02571 | 7.575292 | -5.11219 | 5.13E-05 | 0.000463 | 1.744731 |
| HIST1H2BI | 2.461437 | 6.289922 | 5.106723 | 5.20E-05 | 0.000468 | 1.73221 |
| GTSE1 | 2.398194 | 8.041335 | 5.103117 | 5.24E-05 | 0.00047 | 1.723944 |
| SLMAP | -2.41596 | 8.258345 | -5.10157 | 5.26E-05 | 0.000471 | 1.720393 |
| P2RX6 | -2.305 | 5.939482 | -5.08353 | 5.49E-05 | 0.000487 | 1.679046 |
| EPHA7 | -4.2568 | 8.027105 | -5.07277 | 5.62E-05 | 0.000496 | 1.654357 |
| CBLN2 | -3.44209 | 5.705858 | -5.06477 | 5.73E-05 | 0.000503 | 1.636013 |
| RNF183 | 2.696516 | 7.396725 | 5.061255 | 5.77E-05 | 0.000506 | 1.627936 |
| SAPCD2 | 2.768026 | 11.15529 | 5.057765 | 5.82E-05 | 0.000508 | 1.619927 |
| ZNF536 | -2.26685 | 7.305635 | -5.04567 | 5.99E-05 | 0.00052 | 1.592156 |
| CCNA2 | 2.020542 | 10.42472 | 5.044709 | 6.00E-05 | 0.00052 | 1.589958 |
| CDK1 | 2.817661 | 10.35189 | 5.040008 | 6.07E-05 | 0.000524 | 1.579164 |
| TOX | -2.50164 | 7.397629 | -5.01243 | 6.46E-05 | 0.000553 | 1.515809 |
| ASB9 | 2.038197 | 4.01484 | 4.999189 | 6.67E-05 | 0.000566 | 1.485385 |
| DAND5 | -2.43754 | 6.448828 | -4.99273 | 6.77E-05 | 0.000571 | 1.470541 |
| COL9A1 | 2.5793 | 7.747819 | 4.977827 | 7.00E-05 | 0.000586 | 1.436268 |
| COL6A2 | -2.00023 | 10.81618 | -4.97086 | 7.12E-05 | 0.000593 | 1.420253 |
| MGAM2 | 2.938321 | 5.640986 | 4.959702 | 7.30E-05 | 0.000604 | 1.394575 |
| TIMP4 | -2.4598 | 9.486495 | -4.95735 | 7.34E-05 | 0.000607 | 1.389171 |
| KIF20A | 3.00605 | 8.761479 | 4.947589 | 7.51E-05 | 0.000617 | 1.3667 |
| NKX2-3 | -2.36194 | 10.75212 | -4.94079 | 7.63E-05 | 0.000625 | 1.35106 |
| CHI3L1 | 3.258474 | 7.552812 | 4.930099 | 7.82E-05 | 0.000637 | 1.326437 |
| HIST1H2BJ | 2.163951 | 6.179088 | 4.918397 | 8.04E-05 | 0.000652 | 1.299491 |
| GREM2 | -3.11378 | 7.644164 | -4.91638 | 8.08E-05 | 0.000655 | 1.294845 |
| CHRNA3 | -3.57081 | 7.834053 | -4.90503 | 8.29E-05 | 0.000667 | 1.268699 |
| IL20RA | 2.202632 | 7.197349 | 4.886121 | 8.66E-05 | 0.000691 | 1.225129 |
| CHRNB2 | -2.3813 | 4.225322 | -4.87893 | 8.81E-05 | 0.000701 | 1.208553 |
| CDC6 | 2.341986 | 7.543306 | 4.873881 | 8.91E-05 | 0.000709 | 1.196915 |
| POPDC2 | -3.66075 | 9.453898 | -4.86299 | 9.14E-05 | 0.000722 | 1.171809 |
| RAB23 | -2.14881 | 7.997256 | -4.85948 | 9.22E-05 | 0.000726 | 1.16371 |
| KIF11 | 2.361377 | 7.357086 | 4.853003 | 9.36E-05 | 0.000735 | 1.148772 |
| NEURL3 | 2.345424 | 6.867786 | 4.843148 | 9.57E-05 | 0.000748 | 1.126041 |
| CADPS | 2.265298 | 6.541156 | 4.83144 | 9.84E-05 | 0.000766 | 1.099031 |
| ASB2 | -2.48936 | 8.762206 | -4.81301 | 0.000103 | 0.000793 | 1.056505 |
| ACADL | -2.68375 | 3.489542 | -4.80562 | 0.000104 | 0.000805 | 1.039443 |
| DUSP4 | 2.179919 | 7.971368 | 4.800675 | 0.000106 | 0.000811 | 1.028027 |
| NPSR1 | 2.899697 | 5.924475 | 4.79994 | 0.000106 | 0.000812 | 1.026329 |
| MROH2B | -2.53132 | 3.286144 | -4.79976 | 0.000106 | 0.000812 | 1.025903 |
| COLEC12 | -2.48602 | 7.853373 | -4.78241 | 0.00011 | 0.000838 | 0.985852 |
| TOP2A | 2.945715 | 10.4078 | 4.776546 | 0.000112 | 0.000846 | 0.97231 |
| TRPM6 | -3.41935 | 8.106764 | -4.76832 | 0.000114 | 0.00086 | 0.953302 |
| SLC35D3 | 3.201178 | 6.122386 | 4.757181 | 0.000117 | 0.000876 | 0.927577 |
| AIF1L | -2.12087 | 9.608413 | -4.75386 | 0.000118 | 0.000881 | 0.919911 |
| PKMYT1 | 2.904236 | 8.792844 | 4.748417 | 0.000119 | 0.000889 | 0.907329 |
| RAD51AP1 | 2.200816 | 8.306557 | 4.740193 | 0.000122 | 0.000902 | 0.888323 |
| NUSAP1 | 2.321665 | 8.741427 | 4.73493 | 0.000123 | 0.000909 | 0.876162 |
| WWTR1 | -2.28714 | 12.37215 | -4.71556 | 0.000129 | 0.00094 | 0.831398 |
| CDC25A | 2.446577 | 9.691297 | 4.713134 | 0.00013 | 0.000944 | 0.825779 |
| CRISPLD2 | -2.11645 | 13.01876 | -4.71142 | 0.00013 | 0.000947 | 0.821827 |
| GJC1 | -2.83056 | 10.74384 | -4.70695 | 0.000131 | 0.000953 | 0.811478 |
| ADAMTS14 | 2.05011 | 7.94203 | 4.698696 | 0.000134 | 0.000968 | 0.792397 |
| SLC7A2 | -2.62767 | 8.771828 | -4.69563 | 0.000135 | 0.000974 | 0.785312 |
| BNC2 | -2.17266 | 8.432139 | -4.67207 | 0.000143 | 0.001017 | 0.730811 |
| HTR4 | -3.30176 | 7.192018 | -4.66551 | 0.000145 | 0.001031 | 0.715632 |
| CDCA2 | 2.121021 | 9.228303 | 4.655642 | 0.000148 | 0.00105 | 0.692814 |
| MCMDC2 | 2.006876 | 4.991458 | 4.64023 | 0.000154 | 0.001077 | 0.657154 |
| AKAP12 | -3.0185 | 10.65445 | -4.63477 | 0.000156 | 0.001089 | 0.644515 |
| TENM2 | -2.59528 | 4.140242 | -4.61086 | 0.000165 | 0.001141 | 0.589184 |
| LRP1B | -2.33797 | 3.292098 | -4.60347 | 0.000167 | 0.001158 | 0.572066 |
| DACH1 | 2.0107 | 8.538289 | 4.595869 | 0.00017 | 0.001174 | 0.554479 |
| FERMT1 | 2.840389 | 11.23865 | 4.592597 | 0.000172 | 0.001181 | 0.546904 |
| GRB7 | 2.371947 | 11.677 | 4.589929 | 0.000173 | 0.001187 | 0.540728 |
| SLC26A2 | -3.00599 | 12.76685 | -4.58477 | 0.000175 | 0.0012 | 0.528793 |
| IDO1 | 3.060895 | 9.962211 | 4.583622 | 0.000175 | 0.001201 | 0.526127 |
| DPYSL3 | -2.20927 | 12.26014 | -4.58035 | 0.000177 | 0.001209 | 0.518548 |
| MELK | 2.773838 | 8.105931 | 4.574743 | 0.000179 | 0.001219 | 0.505567 |
| MMP12 | 3.27437 | 11.12494 | 4.566933 | 0.000182 | 0.001237 | 0.487484 |
| AURKB | 2.096248 | 11.26599 | 4.550057 | 0.00019 | 0.001274 | 0.448402 |
| OIP5 | 2.509182 | 8.961778 | 4.542111 | 0.000193 | 0.001295 | 0.430002 |
| SLC6A14 | 3.454322 | 6.363109 | 4.526333 | 0.000201 | 0.001333 | 0.393459 |
| CCDC136 | -2.34108 | 9.825536 | -4.50436 | 0.000211 | 0.00139 | 0.342563 |
| CNTNAP5 | -3.24859 | 3.664193 | -4.4989 | 0.000214 | 0.001403 | 0.329917 |
| RAD54L | 2.559053 | 8.78265 | 4.495534 | 0.000216 | 0.001409 | 0.322117 |
| PRRG3 | -2.63372 | 6.288022 | -4.48809 | 0.000219 | 0.001432 | 0.304865 |
| MAPT | -3.03831 | 4.670919 | -4.48203 | 0.000222 | 0.00145 | 0.290841 |
| AS3MT | -2.35895 | 6.866369 | -4.48029 | 0.000223 | 0.001452 | 0.286809 |
| C6orf222 | 2.848892 | 10.40968 | 4.466449 | 0.000231 | 0.001489 | 0.254736 |
| GPR143 | 3.115906 | 8.729801 | 4.463894 | 0.000232 | 0.001497 | 0.248818 |
| AKR1C4 | 2.835993 | 3.451266 | 4.447585 | 0.000241 | 0.00154 | 0.211033 |
| STAC | -3.12838 | 5.313465 | -4.44151 | 0.000245 | 0.001559 | 0.196961 |
| KIAA0101 | 2.3065 | 7.652785 | 4.439208 | 0.000246 | 0.001564 | 0.191624 |
| CRABP1 | -2.86125 | 7.726263 | -4.4157 | 0.00026 | 0.001628 | 0.137161 |
| SPOCK3 | -2.16395 | 3.835772 | -4.39305 | 0.000274 | 0.001699 | 0.08469 |
| ACSBG1 | -2.36286 | 4.244959 | -4.38346 | 0.00028 | 0.001728 | 0.062468 |
| TTK | 2.920945 | 8.84042 | 4.35922 | 0.000297 | 0.001809 | 0.006308 |
| AGMAT | 2.738247 | 9.776274 | 4.348337 | 0.000304 | 0.001839 | -0.0189 |
| KRTCAP3 | 2.207406 | 8.095521 | 4.332923 | 0.000316 | 0.001895 | -0.05461 |
| CLVS2 | -2.05242 | 3.243005 | -4.31498 | 0.000329 | 0.001958 | -0.09616 |
| HIST2H3C | 2.537351 | 11.13538 | 4.306648 | 0.000336 | 0.001985 | -0.11547 |
| PRDM13 | 3.270856 | 3.823787 | 4.291376 | 0.000348 | 0.002049 | -0.15084 |
| IFNG | 2.681587 | 4.722521 | 4.268867 | 0.000367 | 0.002137 | -0.20296 |
| ARMC4 | -2.09009 | 6.696526 | -4.26079 | 0.000374 | 0.002161 | -0.22167 |
| ZCCHC24 | -2.02995 | 12.63464 | -4.26008 | 0.000375 | 0.002164 | -0.22331 |
| PENK | -3.96766 | 5.138673 | -4.25824 | 0.000376 | 0.00217 | -0.22758 |
| VIPR2 | -2.01094 | 6.153983 | -4.22025 | 0.000411 | 0.002336 | -0.31551 |
| MUM1L1 | -2.41962 | 4.586557 | -4.1956 | 0.000436 | 0.002451 | -0.37255 |
| HIST1H2AM | 2.105996 | 5.13381 | 4.184922 | 0.000447 | 0.002505 | -0.39725 |
| GINS2 | 2.139463 | 11.03654 | 4.18297 | 0.000449 | 0.002512 | -0.40177 |
| NRK | -2.80879 | 6.691379 | -4.17922 | 0.000453 | 0.00253 | -0.41044 |
| CDHR4 | -2.21358 | 3.657911 | -4.17699 | 0.000455 | 0.002537 | -0.4156 |
| AADACL2 | -2.45258 | 3.810721 | -4.16499 | 0.000468 | 0.002593 | -0.44336 |
| DNAH2 | 2.284916 | 5.461525 | 4.158258 | 0.000476 | 0.00263 | -0.45893 |
| CXCL6 | 2.437013 | 3.821839 | 4.139113 | 0.000498 | 0.002725 | -0.50319 |
| CKB | -2.86816 | 15.0374 | -4.12093 | 0.000519 | 0.002816 | -0.54523 |
| KIF26B | 2.485714 | 6.330138 | 4.110964 | 0.000532 | 0.002866 | -0.56825 |
| CCNB2 | 2.56085 | 10.25332 | 4.103742 | 0.000541 | 0.002907 | -0.58494 |
| KIAA1462 | -2.28909 | 10.63846 | -4.07112 | 0.000584 | 0.00309 | -0.66027 |
| CORIN | 2.478254 | 5.063559 | 4.05013 | 0.000614 | 0.003218 | -0.70874 |
| HBB | -2.20868 | 14.00439 | -4.04457 | 0.000622 | 0.003248 | -0.72157 |
| RTKN2 | 2.532399 | 6.788354 | 4.042526 | 0.000625 | 0.003261 | -0.72628 |
| IQSEC3 | -2.15437 | 5.819849 | -4.03457 | 0.000636 | 0.003307 | -0.74464 |
| RIPPLY2 | -2.52335 | 4.093406 | -4.0218 | 0.000656 | 0.003387 | -0.77409 |
| IL17RB | 2.366456 | 10.57845 | 3.999352 | 0.000691 | 0.003544 | -0.82585 |
| BIK | 2.346428 | 10.97898 | 3.971732 | 0.000738 | 0.003736 | -0.8895 |
| ASB5 | -5.10056 | 6.063344 | -3.96309 | 0.000753 | 0.003793 | -0.90941 |
| LRRIQ4 | 2.870573 | 4.299744 | 3.952204 | 0.000772 | 0.003868 | -0.93446 |
| ZWINT | 2.306255 | 9.248782 | 3.918629 | 0.000836 | 0.00413 | -1.01172 |
| WDR17 | -2.61374 | 5.624257 | -3.89043 | 0.000893 | 0.004354 | -1.07655 |
| SFRP2 | -3.18886 | 10.48445 | -3.88815 | 0.000898 | 0.004372 | -1.08178 |
| RASSF10 | 2.667597 | 5.94413 | 3.873907 | 0.000928 | 0.00449 | -1.1145 |
| CYP1B1 | -2.13247 | 9.779291 | -3.86712 | 0.000943 | 0.004546 | -1.13008 |
| POLQ | 2.407513 | 8.146028 | 3.856035 | 0.000968 | 0.004646 | -1.15552 |
| FBXO32 | -2.49352 | 10.39767 | -3.85178 | 0.000978 | 0.004685 | -1.16528 |
| HIST1H2AL | 2.478401 | 6.644291 | 3.850843 | 0.00098 | 0.004694 | -1.16744 |
| SAA4 | 3.140951 | 4.485266 | 3.849779 | 0.000982 | 0.004697 | -1.16988 |
| REG1A | 5.145209 | 9.896006 | 3.844843 | 0.000994 | 0.004735 | -1.1812 |
| HIST1H3F | 2.179524 | 8.425575 | 3.840257 | 0.001005 | 0.004779 | -1.19172 |
| HIST1H2AI | 2.522036 | 8.889322 | 3.78974 | 0.001131 | 0.005258 | -1.30747 |
| PCSK9 | 2.864499 | 10.90214 | 3.778373 | 0.001161 | 0.00536 | -1.33349 |
| CD70 | 2.431305 | 7.121721 | 3.778093 | 0.001162 | 0.005362 | -1.33413 |
| GDNF-AS1 | -2.02679 | 4.015412 | -3.77766 | 0.001163 | 0.005363 | -1.33511 |
| KRT25 | -2.1785 | 3.018868 | -3.73849 | 0.001275 | 0.005772 | -1.42466 |
| KLK10 | 3.245902 | 3.740566 | 3.737824 | 0.001277 | 0.005779 | -1.42618 |
| GJB3 | 2.800893 | 10.85717 | 3.721605 | 0.001327 | 0.005959 | -1.46321 |
| PNCK | -2.00014 | 6.207238 | -3.67631 | 0.001475 | 0.006483 | -1.56648 |
| RAD51 | 2.10822 | 6.929454 | 3.67361 | 0.001484 | 0.006515 | -1.57262 |
| SPIB | -3.37349 | 7.186214 | -3.66885 | 0.001501 | 0.006563 | -1.58346 |
| CDC45 | 2.87277 | 9.307993 | 3.65569 | 0.001548 | 0.006743 | -1.6134 |
| IP6K3 | -2.16698 | 6.908838 | -3.65278 | 0.001558 | 0.006782 | -1.62001 |
| HNF1A | 2.151978 | 10.5994 | 3.640034 | 0.001605 | 0.006963 | -1.649 |
| KIAA1257 | 2.044764 | 3.48037 | 3.634779 | 0.001625 | 0.007027 | -1.66094 |
| HS6ST2 | 2.195621 | 7.19647 | 3.627578 | 0.001653 | 0.00713 | -1.6773 |
| PIFO | -2.40939 | 5.913131 | -3.61703 | 0.001694 | 0.00727 | -1.70126 |
| NMU | 2.386333 | 7.470042 | 3.5904 | 0.001803 | 0.007633 | -1.76165 |
| PVRL4 | 2.863241 | 8.815968 | 3.580463 | 0.001845 | 0.007778 | -1.78417 |
| CEND1 | -2.17296 | 4.632149 | -3.5593 | 0.001938 | 0.008073 | -1.83208 |
| PNPLA3 | 2.65044 | 5.927929 | 3.551312 | 0.001975 | 0.008207 | -1.85014 |
| HOXD10 | -2.29103 | 8.374899 | -3.49939 | 0.002228 | 0.008998 | -1.96734 |
| C4BPA | 2.015566 | 7.221017 | 3.473991 | 0.002364 | 0.00947 | -2.02454 |
| PLEK2 | 2.158291 | 12.02646 | 3.441373 | 0.00255 | 0.010074 | -2.09784 |
| PCK1 | -2.13712 | 11.909 | -3.4142 | 0.002716 | 0.01058 | -2.15877 |
| HACD1 | -2.18675 | 7.510243 | -3.40129 | 0.002798 | 0.010815 | -2.18766 |
| FUT9 | -2.51874 | 3.132177 | -3.39976 | 0.002808 | 0.010843 | -2.19108 |
| IL1A | 2.870177 | 5.066154 | 3.398956 | 0.002813 | 0.010856 | -2.19289 |
| CPB1 | -2.96505 | 7.649143 | -3.38843 | 0.002883 | 0.011058 | -2.21642 |
| STON1-GTF2A1L | -2.67353 | 5.538427 | -3.34724 | 0.003171 | 0.011964 | -2.30835 |
| CHST4 | 2.941752 | 5.117405 | 3.336464 | 0.003251 | 0.012195 | -2.33236 |
| NR4A3 | -2.87057 | 10.09032 | -3.32622 | 0.003328 | 0.012399 | -2.35516 |
| GCG | -3.21087 | 8.492638 | -3.28319 | 0.003675 | 0.013441 | -2.45067 |
| OLR1 | 2.597775 | 4.108544 | 3.281663 | 0.003688 | 0.013484 | -2.45407 |
| CCIN | -2.13706 | 4.456059 | -3.27035 | 0.003786 | 0.013759 | -2.47912 |
| UGT2B15 | 2.324054 | 3.808313 | 3.233303 | 0.004122 | 0.014709 | -2.56097 |
| KRTAP13-2 | -3.74458 | 5.996178 | -3.2287 | 0.004166 | 0.014824 | -2.57112 |
| MKI67 | 2.097296 | 10.66815 | 3.222676 | 0.004224 | 0.014967 | -2.5844 |
| GAS1 | -2.62654 | 8.57433 | -3.21028 | 0.004346 | 0.015275 | -2.6117 |
| CA9 | 2.226311 | 10.24153 | 3.204875 | 0.0044 | 0.015433 | -2.62359 |
| CHGA | -4.64547 | 12.07538 | -3.15022 | 0.004986 | 0.017111 | -2.74348 |
| KCNT1 | -2.38283 | 4.84388 | -3.0969 | 0.005631 | 0.01893 | -2.85975 |
| NEU4 | -2.20237 | 11.14111 | -3.07548 | 0.005912 | 0.019628 | -2.90629 |
| TRIM31 | 2.379629 | 9.241956 | 3.059253 | 0.006134 | 0.020154 | -2.94145 |
| ARC | -2.93168 | 7.480062 | -2.99656 | 0.007069 | 0.022663 | -3.07669 |
| EPYC | 2.394423 | 3.301063 | 2.958677 | 0.007699 | 0.024313 | -3.15789 |
| MEDAG | -2.0289 | 9.156105 | -2.9451 | 0.007938 | 0.024787 | -3.1869 |
| KIF12 | 2.093506 | 10.70724 | 2.940939 | 0.008013 | 0.024947 | -3.19578 |
| LCN2 | 2.51792 | 12.909 | 2.934613 | 0.008128 | 0.025219 | -3.20927 |
| CXCL2 | 2.498094 | 10.63602 | 2.93221 | 0.008172 | 0.025314 | -3.21439 |
| MB | -2.00321 | 9.12369 | -2.92602 | 0.008286 | 0.025614 | -3.22757 |
| IYD | 2.069375 | 7.793626 | 2.898836 | 0.008807 | 0.026983 | -3.28534 |
| NKAIN2 | -2.42414 | 4.696717 | -2.8816 | 0.009153 | 0.027828 | -3.32186 |
| CXCL13 | 3.301457 | 9.023068 | 2.877976 | 0.009228 | 0.027966 | -3.32952 |
| HBG1 | -2.15492 | 5.335852 | -2.87707 | 0.009246 | 0.028016 | -3.33144 |
| NIPAL4 | -2.36925 | 7.032914 | -2.85064 | 0.009808 | 0.029372 | -3.38723 |
| WNT4 | 2.044192 | 6.194499 | 2.820266 | 0.010495 | 0.03103 | -3.45106 |
| MT1A | -2.2615 | 10.25931 | -2.81934 | 0.010516 | 0.031062 | -3.45299 |
| ALDOB | 2.270513 | 3.885105 | 2.805725 | 0.010839 | 0.031792 | -3.48151 |
| C3orf80 | -2.17876 | 6.795636 | -2.73763 | 0.012602 | 0.03593 | -3.62323 |
| PTF1A | 2.08189 | 3.170032 | 2.721958 | 0.013044 | 0.036877 | -3.65562 |
| CCL2 | -2.08389 | 11.33545 | -2.71786 | 0.013162 | 0.037083 | -3.66407 |
| CXCL8 | 2.707102 | 7.126727 | 2.639982 | 0.01561 | 0.042388 | -3.82365 |
| SMPX | -4.12555 | 7.170312 | -2.61238 | 0.016576 | 0.044376 | -3.87966 |
| STMN2 | -2.23235 | 8.541298 | -2.56567 | 0.018341 | 0.048084 | -3.97382 |
| SLC13A2 | -2.61643 | 7.239061 | -2.55055 | 0.018949 | 0.049413 | -4.0041 |

Supplementary table 4. The differentially expressed miRNAs (diff-miRNAs)

| Nodes | Description | Degree |
| --- | --- | --- |
| hsa-miR-497-5p | down-miRNA | 62 |
| hsa-miR-31-5p | up-miRNA | 34 |
| hsa-miR-139-5p | down-miRNA | 33 |
| hsa-miR-224-5p | up-miRNA | 25 |
| hsa-miR-1244 | up-miRNA | 25 |
| hsa-miR-188-5p | up-miRNA | 22 |
| RP11-307B6.3 | down-lncRNA | 18 |
| RP11-305O6.3 | down-lncRNA | 17 |
| hsa-miR-19b-1-5p | up-miRNA | 16 |
| MIR143HG | down-lncRNA | 16 |
| hsa-miR-764 | up-miRNA | 13 |
| RP11-6O2.3 | down-lncRNA | 10 |
| AF001548.3 | down-lncRNA | 9 |
| AP000892.6 | down-lncRNA | 8 |
| DYNC1I1 | down-gene | 6 |
| C1orf95 | down-gene | 6 |
| JPH2 | down-gene | 6 |
| MYLK | down-gene | 6 |
| GNAO1 | down-gene | 6 |
| hsa-miR-452-3p | up-miRNA | 5 |
| ONECUT2 | up-gene | 5 |
| FAM84B | up-gene | 5 |
| AR | down-gene | 5 |
| SNCA | down-gene | 5 |
| DPYSL5 | down-gene | 5 |
| SLC2A4 | down-gene | 5 |
| LRCH2 | down-gene | 5 |
| TRIM9 | down-gene | 4 |
| KIAA1462 | down-gene | 4 |
| CNTN2 | down-gene | 4 |
| CNTNAP5 | down-gene | 4 |
| DMD | down-gene | 4 |
| SLC35F1 | down-gene | 4 |
| FGFR1 | down-gene | 4 |
| RP11-474D1.3 | up-lncRNA | 3 |
| ZNF483 | down-gene | 3 |
| TRPC1 | down-gene | 3 |
| SIM2 | up-gene | 3 |
| RAB6B | down-gene | 3 |
| NBEA | down-gene | 3 |
| hsa-miR-145-3p | down-miRNA | 3 |
| KCNA1 | down-gene | 3 |
| TRIM59 | up-gene | 3 |
| MEIS2 | down-gene | 3 |
| MAP1B | down-gene | 3 |
| AC007392.3 | down-lncRNA | 3 |
| SLMAP | down-gene | 3 |
| SGCA | down-gene | 3 |
| RASSF8 | down-gene | 3 |
| JAZF1 | down-gene | 3 |
| FERMT2 | down-gene | 3 |
| RP11-679B19.1 | down-lncRNA | 2 |
| LA16c-60D12.2 | up-lncRNA | 2 |
| LINC00473 | down-lncRNA | 2 |
| RP11-728F11.4 | down-lncRNA | 2 |
| RP11-734K21.5 | up-lncRNA | 2 |
| RP11-1069G10.1 | down-lncRNA | 2 |
| ANK2 | down-gene | 2 |
| ADCYAP1R1 | down-gene | 2 |
| RGMA | down-gene | 2 |
| PRICKLE2 | down-gene | 2 |
| ITPKB | down-gene | 2 |
| TMOD2 | down-gene | 2 |
| SLC7A2 | down-gene | 2 |
| SCN9A | down-gene | 2 |
| MKI67 | up-gene | 2 |
| KIF5C | down-gene | 2 |
| COLGALT2 | down-gene | 2 |
| NR4A3 | down-gene | 2 |
| PCDH9 | down-gene | 2 |
| GSTM3 | down-gene | 2 |
| FBXO41 | up-gene | 2 |
| FBXO32 | down-gene | 2 |
| CHRNB2 | down-gene | 2 |
| SLC26A2 | down-gene | 2 |
| PEG3 | down-gene | 2 |
| NGFR | down-gene | 2 |
| MUM1L1 | down-gene | 2 |
| CSRNP3 | down-gene | 2 |
| CHRDL1 | down-gene | 2 |
| AP1S2 | down-gene | 2 |
| TOX | down-gene | 2 |
| PRIMA1 | down-gene | 2 |
| PPP2R3A | down-gene | 2 |
| NKAIN2 | down-gene | 2 |
| PTGER3 | down-gene | 2 |
| AC074011.2 | down-lncRNA | 2 |
| EPHA7 | down-gene | 2 |
| C20orf194 | down-gene | 2 |
| RP11-266K4.13 | down-lncRNA | 1 |
| SERTAD4-AS1 | down-lncRNA | 1 |
| AC123023.1 | up-lncRNA | 1 |
| RP11-706O15.3 | up-lncRNA | 1 |
| RP5-1056H1.2 | up-lncRNA | 1 |
| RP11-554A11.4 | down-lncRNA | 1 |
| RP11-396O20.2 | down-lncRNA | 1 |
| RP11-1149O23.4 | up-lncRNA | 1 |
| RP11-1334A24.5 | down-lncRNA | 1 |
| PCAT7 | up-lncRNA | 1 |
| RP11-108K3.2 | up-lncRNA | 1 |
| LL22NC03-N64E9.1 | up-lncRNA | 1 |
| RP11-73M7.1 | up-lncRNA | 1 |
| AL163953.2 | up-lncRNA | 1 |
| CTD-2116N20.1 | up-lncRNA | 1 |
| RP11-150O12.3 | up-lncRNA | 1 |
| RP11-15F12.1 | up-lncRNA | 1 |
| RP11-79H23.3 | up-lncRNA | 1 |
| MPPED2 | down-gene | 1 |
| ZNF536 | down-gene | 1 |
| SYT4 | down-gene | 1 |
| SYNGR1 | down-gene | 1 |
| SVEP1 | down-gene | 1 |
| SNCG | down-gene | 1 |
| SLC7A5 | up-gene | 1 |
| SLC6A14 | up-gene | 1 |
| SALL4 | up-gene | 1 |
| RNF43 | up-gene | 1 |
| REEP1 | down-gene | 1 |
| PRDM12 | up-gene | 1 |
| MACC1 | up-gene | 1 |
| LRP1B | down-gene | 1 |
| KIF23 | up-gene | 1 |
| KCNN4 | up-gene | 1 |
| ITGA2 | up-gene | 1 |
| IP6K3 | down-gene | 1 |
| HTR4 | down-gene | 1 |
| FERMT1 | up-gene | 1 |
| E2F7 | up-gene | 1 |
| DSEL | down-gene | 1 |
| DACH1 | up-gene | 1 |
| CYP2S1 | up-gene | 1 |
| CYP1B1 | down-gene | 1 |
| CHD5 | down-gene | 1 |
| CDC25A | up-gene | 1 |
| AXIN2 | up-gene | 1 |
| ACSBG1 | down-gene | 1 |
| GPR12 | down-gene | 1 |
| EPYC | up-gene | 1 |
| PRKAA2 | down-gene | 1 |
| NTRK3 | down-gene | 1 |
| MCMDC2 | up-gene | 1 |
| HSD17B6 | down-gene | 1 |
| HLF | down-gene | 1 |
| DPY19L2 | down-gene | 1 |
| DAND5 | down-gene | 1 |
| CXCL9 | up-gene | 1 |
| CTNND2 | down-gene | 1 |
| CLDN1 | up-gene | 1 |
| CDC6 | up-gene | 1 |
| CD1A | up-gene | 1 |
| PHOX2B | down-gene | 1 |
| HOXD10 | down-gene | 1 |
| FUT9 | down-gene | 1 |
| CRTAP | down-gene | 1 |
| SUSD5 | down-gene | 1 |
| RBMS3 | down-gene | 1 |
| PBX1 | down-gene | 1 |
| KLB | down-gene | 1 |
| WASF3 | down-gene | 1 |
| SFRP5 | down-gene | 1 |
| MMP1 | up-gene | 1 |
| GNG7 | down-gene | 1 |
| GAS1 | down-gene | 1 |
| CRABP1 | down-gene | 1 |
| GRIK2 | down-gene | 1 |
| DIO2 | up-gene | 1 |
| TOP2A | up-gene | 1 |
| PDE3A | down-gene | 1 |
| NRK | down-gene | 1 |
| MAD2L1 | up-gene | 1 |
| LRFN5 | down-gene | 1 |
| ITIH5 | down-gene | 1 |
| IL20RA | up-gene | 1 |
| EBF1 | down-gene | 1 |
| CMYA5 | down-gene | 1 |
| C6orf222 | up-gene | 1 |
| VN1R1 | down-gene | 1 |
| SLC26A7 | down-gene | 1 |
| SLC17A8 | down-gene | 1 |
| PTN | down-gene | 1 |
| PARD6B | up-gene | 1 |
| JAM2 | down-gene | 1 |
| HBB | down-gene | 1 |
| FGFBP2 | down-gene | 1 |
| CHRNA3 | down-gene | 1 |
| CADM3 | down-gene | 1 |
